# A synonymous variant contributes to a rare Wiedemann-Rautenstrauch syndrome complicated with mild anemia *via* affecting pre-mRNA splicing

**DOI:** 10.3389/fnmol.2022.1026530

**Published:** 2022-10-28

**Authors:** Qiongling Peng, Yan Zhang, Binqiang Xian, Lianying Wu, Jianying Ding, Wuwu Ding, Xin Zhang, Bilan Ding, Ding Li, Jin Wu, Xiaowu Hu, Guanting Lu

**Affiliations:** ^1^Department of Child Healthcare, Shenzhen Baoan Women's and Children's Hospital, Jinan University, Shenzhen, China; ^2^Department of Obstetrics and Gynecology, Strategic Support Force Medical Center, Beijing, China; ^3^Laboratory of Translational Medicine Research, Deyang People’s Hospital, Deyang, China; ^4^Deyang Key Laboratory of Tumor Molecular Research, Department of Pathology, Deyang People’s Hospital, Deyang, China; ^5^Clinical Laboratory, Sichuan Provincial Rehabilitation Hospital, Chengdu, China

**Keywords:** Wiedemann-Rautenstrauch syndrome, Fanconi anemia, whole-exome sequencing, POLR3A, FANCA, pre-mRNA splicing

## Abstract

Wiedemann-Rautenstrauch syndrome (WDRTS) is an extremely rare autosomal recessive neonatal disorder. Currently, over 50 cases with variable phenotypes of WDRTS have been reported. In our cohort of prenatal and postnatal growth retardation, a female proband was found to have general growth retardation, neurocutaneous syndrome, and anemia. Karyotype test and array-CGH detected no obvious chromosomal aberrations. Trio-based whole-exome sequencing (Trio-WES) identified bi-allelic compound mutations in the coding sequence (CDS) of *POLR3A* gene (c.3342C > T, p.Ser1114 = and c.3718G > A, p.Gly1240Ser). For the mild anemia phenotype, the underlying causal genetic factors could be attributed to the compound heterozygous mutations in *FANCA* gene (c.2832dup, p.Ala945CysfsTer6 and c.1902 T > G, p.Asp634Glu). Mini-gene reporter assays revealed that the synonymous variant of *POLR3A* and the missense variant of *FANCA* could affect pre-mRNA splicing of each gene. For *POLR3A*, the synonymous mutation (c.3342C > T, p.Ser1114=) generated three types of aberrant isoforms. Therefore, the female patient was finally diagnosed as WDRTS caused by *POLR3A*. For *FANCA*, the missense variant (c.1902 T > G, p.Asp634Glu) disrupted the normal splicing between exon 21 and 22, and produced two types of abnormal isoforms, one carrying the 1902G and the other spliced between exon 21 and 23 to exclude exon 22. Network analysis showed that POLR3A and FANCA could be STRINGed, indicating both proteins might collaborate for some unknown functions. Current investigation would broaden the knowledge for clinicians and genetic counselors and remind them to interpret those synonymous or predicted “benign” variants more carefully.

## Introduction

Wiedemann-Rautenstrauch syndrome (WDRTS, OMIM#264090), an extremely rare progeroid disorder, was initially reported in two sisters by Rautenstrauch in 1977 (Rautenstrauch and Snigula, 1977). It was characterized by multiple distinct clinical features such as intrauterine growth retardation (IUGR), a progeroid appearance, lipodystrophy, failure to thrive, short stature, hypotonia, prominent scalp veins, teeth abnormalities and variable mental impairment (Toriello, 1990; Pivnick et al., 2000; Paolacci et al., 2017). In 1979, Wiedemann and Rautenstrauch considered this distinct neonatal progeroid syndrome to be transmitted under an autosomal recessive (AR) inheritance mode (Wiedemann, 1979).

Till now, over 50 individuals with variable phenotypes of WDRTS have been reported (Paolacci et al., 2017). Homozygous or bi-allelic heterozygous mutations of RNA polymerase III subunit A (*POLR3A*, OMIM#614258) were proved to be the causal for WDRTS (Jay et al., 2016; Paolacci et al., 2018; Wambach et al., 2018; Temel et al., 2020). A few other cases with neonatal-onset progeria and lipodystrophy were identified to be caused by mutations in fibrillin 1 (*FBN1*, OMIM#134797; Graul-Neumann et al., 2010; Garg and Xing, 2014), caveolin 1 (*CAV1*,OMIM#601047; Garg et al., 2015; Schrauwen et al., 2015), catalytic subunit of DNA polymerase delta 1 (*POLD1*, OMIM#174761; Elouej et al., 2017; Sasaki et al., 2018) and solute carrier family 25 member 24 (*SLC25A24*, OMIM#608744; Ehmke et al., 2017; Rodríguez-García et al., 2018). Since neonatal-onset progeria and lipodystrophy were also core clinical phenotypes of WDRTS, it would pose a big challenge to discriminate WDRTS from other neonatal-onset progeria and lipodystrophy disorders in the early period, and to give appropriate and timely symptomatic treatments.

Fanconi Anemia (FA, OMIM#227650) was a group of well-known clinically and genetically heterogeneous disorders (Bogliolo and Surralles, 2015), and characterized by distinct clinical features including developmental abnormalities in major organ systems, early-onset bone marrow failure, cellular sensitivity to DNA crosslinking agents, and a high predisposition to cancer (Nepal et al., 2017). The prevalence of FA was estimated at 1–5 in 1,000,000 live births (D’Andrea, 2010; Kottemann and Smogorzewska, 2013). It had been reported that FA could be caused by autosomal biallelic germline inactivation of any one of the 22 genes (*FANCA*-*FANCW*), except for the X-chromosomal *FANCB* gene (Niraj et al., 2019). Mutations in *FANCA* (OMIM#607139), *FANCC* (OMIM#613899) and *FANCG* (OMIM#602956) genes accounted for 60 ~ 65%, ~15% and ~10% of all the reported FA cases, respectively (D’Andrea and Grompe, 2003; Dimishkovska et al., 2018; Repczynska et al., 2022).

In our clinic, a 3-years-old female patient was presumptively diagnosed as general growth retardation, neurocutaneous syndrome, left hip dysplasia and anemia. Later, she was diagnosed as WDRTS according to the clinical phenotypes and bi-allelic mutations in *POLR3A* gene detected by Trio-based whole exome sequencing (trio-WES). Besides, two heterozygous mutations were also detected in *FANCA* gene, resulted in a mild form of Fanconi Anemia. It is worth noting that one benign variant was identified in each gene and confirmed to affect proper pre-mRNA splicing to generate abnormal transcripts.

To our knowledge, this was the first report for a WDRTS complicated with the occurrence of another recessive disorder, Fanconi Anemia (FA). It would broaden the molecular knowledge about WDRTS to clinicians and genetic counselors and reminded them to be more careful for analyzing genetic data and other relevant laboratory results.

## Materials and methods

### Sample collection

This study was conducted in accordance with the Code of Ethics of the World Medical Association (Declaration of Helsinki) for experiments involving humans. This study was approved by the Ethical Committee of the Shenzhen Bao’an Women’s and Children’s Hospital and Deyang People’s Hospital. Written informed consents were obtained from the female’s parent.

Peripheral venous blood was collected from the proband and her parent. Genomic DNA was extracted using the TIANamp Blood DNA Kit (DP348, Tiangen Biotech, Beijing, China) according to the manufacturer’s instructions.

### Array-comparative genomic hybridization

Oligonucleotide Array-comparative genomic hybridization (array-CGH) was performed using the Fetal DNA Chip (Version 1.2) designed by the Chinese University of Hong Kong (CUHK) (Huang et al., 2014). The chip contains a total of 60,000 probes for more than 100 diseases caused by known microduplication/microdeletions. It does not include small fragment chromosomal abnormalities, copy number polymorphism, chimerism and chromosomal rearrangement (Iafrate et al., 2004). The experimental procedures were performed according to the standard Agilent protocol [Agilent Oligonucleotide Array-Based CGH (Array-CGH) for Genomic DNA Analysis, version 3.5]. Hybridized slides were scanned with SureScan High-Resolution Microarray Scanner (G2505B, Agilent Technologies), and the image data were extracted and converted to text files using Agilent Feature Extraction software. The data were graphed and analyzed using Agilent CGH Analytics software.

### Trio-based whole exome sequencing

To investigate the genetic cause of the disease, whole-exome sequencing (WES) was performed for the trio at MyGenostics. Briefly, the fragmented genomic DNAs were ligated with the 3′ end of the Illumina adapters and amplified by polymerase chain reaction (PCR). The amplified DNA was captured with Gencap Human whole Exon Kit (52 M) at MyGenostics. The capture procedure was performed in accordance with the manufacturer’s protocol. Finally, the generated libraries were sequenced on Illumina HiSeq 2,500 platform for paired-end sequencing. The sequencing depth was about 100x for each sample.

The analysis of the WES data was carried out according to our previous reports (Lu et al., 2021, 2022). Briefly, clean reads were obtained after removal of adaptors and low-quality reads (multiple Ns and shorter than 40 bp) by Cutadapt (version 1.16) from raw data in fastq format (Kechin et al., 2017). The trimmed clean reads were aligned to the human reference genome (UCSC hg19) using BWA software (version 0.7.10) (Li and Durbin, 2010). The obtained files would be converted to bam format by SAMtools (version 1.2) (Li et al., 2009) and then filtered by BamTools (version 2.4.0) (Barnett et al., 2011). GATK (Genome Analysis Toolkit, version 4.0.8.1) was used to remove duplicated reads (by GATK/MarkDuplicates.jar), to recalibrate bases (by GATK/BaseRecalibrator.jar), and to obtain new bam files (by GATK/ApplyBQSR.jar) for subsequent variant calling by HaplotypeCaller (Auwera et al., 2013). Functional annotation for the GATK-called variants was performed by ANNOVAR (version 2018-04-16; Wang et al., 2010). Variants with a minor allele frequency (MAF) > 1% in the 1,000 Genome Project, or in-house data were removed. Synonymous single nucleotide variants (SNVs) were also removed. SNVs that caused splicing, frameshift, stopgain, or stoploss were retained for subsequent analysis. A position was called as heterozygous if 25% or more of the reads identified the minor allele. The location, type, conservation of the identified variants was obtained from several public databases, such as UCSC Genome Browser, NCBI dbSNP, NCBI ClinVar, 1000Genome, ExAC, TOPMED, gnomAD and gnomAD_exomes. Nonsynonymous SNVs were submitted to PolyPhen-2 (Polymorphism Phenotyping v2; Adzhubei et al., 2013) and PROVEAN (Protein Variation Effect Analyzer; Choi and Chan, 2015) for functional prediction. The pathogenicity of identified variants were also annotated according to the guidelines of American College of Medical Genetics (ACMG) (Riggs et al., 2020). The selected variants were confirmed by Sanger sequencing with an ABI3730xl sequencer (Applied Biosystems, Waltham, Massachusetts, United States). The possibility of identified variant for aberrant splicing was analyzed by SpliceAI (version 1.3.1)^1^ under default settings (Jaganathan et al., 2019).

### Molecular analysis for the identified mutations

The protein sequences of POLR3A and FANCA were downloaded from NCBI GenBank, including 3 primates (*Homo sapiens*, *Pan troglodytes*, and *Macaca mulatta*), 1 cattle (*Bos taurus*), 2 rodents (*Mus musculus*, and *Rattus norvegicus*), 1 Chiroptera (*Artibeus jamaicensis*), 1 bird (*Gallus gallus*), 2 amphibians (*Bufo bufo* and *Xenopus tropicalis*), 2 fishes (*Danio rerio* and *Nothobranchius furzeri*). The protein sequences were aligned by the built-in ClustalW alignment algorithms of MEGA 11 (Gap opening penalty and Gap extension penalty for pairwise alignment and multiple alignment were set as 10.00, 0.10 and 10.00, 0.20, respectively; the Delay divergent cutoff was 30%). The effects of missense mutations on the structural changes were analyzed by the Missense3D and visualized using 3D View.^2^ The gene expressions were evaluated according to the normalized signal intensity of probe 227872_at for POLR3A and 236976_at for FANCA, which were extracted from a gene atlas of human protein-encoding transcriptomes for 79 human tissues (NCBI GEO#GSE1133; Su et al., 2004). The protein interaction network with POLR3A and FANCA was generated by STRING (version 11.5) under default settings. Gene Ontology (GO) analysis was performed for the 10 members of the network under default parameters in the Gene Ontology knowledgebase.^3^

### Mini-gene reporter assays

The genomic regions containing the two mutations (c.3342C > T for *POLR3A* and c.1902 T > G for *FANCA*) were synthesized and cloned into the multiple cloning site (MCS) of pEGFP-N1 plasmid for minigene splicing reporter assays to test their effects on pre-mRNA splicing. As for c.3342C > T of *POLR3A*, the 1,646 bp genomic DNA spanning exon 25 to exon 27 (10:79,742,411-79,744,056, hg19) was cloned into the MCS of pEGFP-N1. For c.1902 T > G of *FNACA*, the 4,135 bp genomic DNA from exon 21 to exon 23 (16:89,838,089-89,842,223, hg19) was cloned into the MCS of pEGFP-N1. The two mutations were introduced by site-directed mutagenesis.

The human embryonic kidney 293 cells (HEK293) or HeLa cells were cultured in high glucose DMEM medium (FI101-01, TransGen, Beijing, China) supplied with 5% fetal bovine sera (FBS) in 5% CO_2_. The constructs were transfected into HEK293 or HeLa cells by *TransIntro* EL/PL Transfection Reagent (FT231-02, TransGen) according to the manufacturer’s protocol. 24 h after transfection, cells were harvested, and lysed by adding 5 ml TransZol (ET101-01, TransGen). The wild-type (WT) and mutated (Mut) constructs were transfected into cells, respectively. Total RNAs were extracted and reversely transcribed into complementary DNAs (cDNAs) by TransScript Reverse Transcriptase (AT101-02, TransGen). The cDNAs were amplified by polymerase chain reaction (PCR) with paired primers (Supplementary Table S1), electrophoresed with agarose gel (1.5%, 120 V for 25 min), and then visualized by ChemiDoc XRS+ Gel Imaging System (Bio-Rad, Hercules, California, United States). DNAs of the bands were extracted and sequenced with an ABI3730xl sequencer (Applied Biosystems, United States).

### Single cell gel electrophoresis assay

The single cell gel electrophoresis (SCGE) assay was performed as previously described with minor modifications (Li et al., 2014; Ji et al., 2018). After separated from 0.5 ml peripheral blood, lymphocytes were washed and resuspended at a density of 10^5^ cells/mL in phosphate-buffered saline (PBS). 30 ml lymphocyte suspension were added in 70 μl of 0.75% low-melting-point agarose. The cell/agarose mixture was added onto the CometSlides which were precoated with 300 μl normal-melting-point agarose (0.75%) and was covered by a coverslip. After solidification, the coverslips were removed from the CometSlides. The CometSlides were submersed in cold fresh alkaline lysis solution for 1.5 h at 4°C. After lysis, the slides were electrophoresed at 30 V for 20 min in a horizontal tank which was filled with cold TBE buffer. Then, the slides were submerged in neutralization buffer for 20 min and stained with ethidium bromide (EB) in darkroom. The comets were observed using a digital fluorescence microscope (ECLIPSE 90i, Nikon, Tokyo, Japan), and images of 200 comets collected for each sample. The comets were analyzed by CASP (Comet Assay Software Project) software. The percentages of DNA in the comet head (HeadDNA%), DNA in the comet tail (TailDNA%), tail length (pix), tail moment (TM) and Olive tail moment (OTM) were calculated to evaluate the DNA damage of lymphocytes.

### Statistical analysis

The statistical analysis was conducted using the SPSS software (version 13) with Student’s t test for the mitomycin C-induced chromosome stress assay, and SCGE assay. *p* value less than 0.05 was considered as significance.

## Results

### Patient description

**Figure 1:**
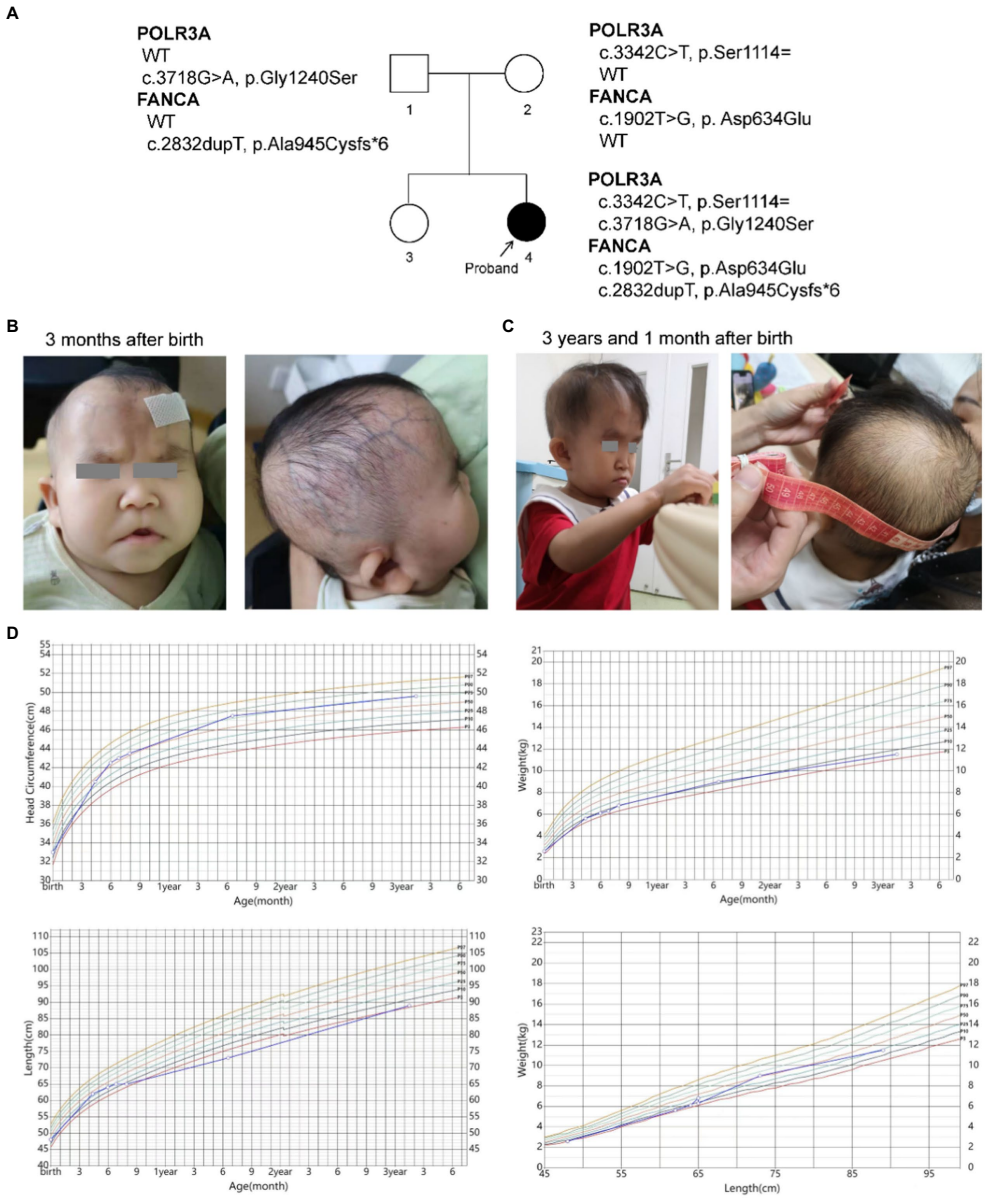
Descriptions of the proband. **(A)** Pedigree of the proband; **(B,C)** Facial and skin features of the proband; **(D)** Growth curves of the proband.

The female proband (46, XX) was born naturally to a non-consanguineous couple in 2019. She has one unaffected healthy elder sister (Figure 1A). Her gestational period was 40^+3^ weeks. Her birth weight was 2.59 kg (P3). Her head occipitofrontal circumference (OFC) and body length at birth were 33 cm (P11) and 48 cm (P14), respectively. At the age of three, her weight, height and OFC were 11.5 kg (P6), 89.0 cm (P4), and 49.6 cm (P76), respectively (Figure 1D). Her mother accepted all regular inspections as required during her pregnancy. No abnormalities were found except for intrauterine growth retardation (IUGR) at 36 weeks of gestation. Her mother had no history of smoking or exposure to harmful hazards during pregnancy. She was breast fed in the first 6 months after birth. Mild feeding difficulty and sucking weakness were observed during that period. After 6 months of age, she gradually established a normal daily diet, but had persistent poor postnatal growth.

#### Manifestations of progeroid phenotypes of WDRTS

The patient had presented progeroid appearance, with sparse scalp hair, poorly developed teeth, and thin subcutaneous fat (Figures 1B,C). Facial dysmorphic features were observed, such as triangular face, prominent forehead with frontal bossing, prominent scalp veins, sparse and broad eyebrows, deep set and long spaced eyes, pinched nose, small mouth with downturned corners, high-arched palate, malformed and low-set ears, and pointed chin. At birth, the patient had two natal teeth in the upper jaw and a gingival cyst of mucous gland in the lower jaw. At 6 months of age, the two natal teeth were removed by a dentist. No new teeth had grown at the same positions up to date.

She was found mild neurodevelopmental delay and hypermyotonia at 3 months of age and received rehabilitation which lasted for 5 months till she could creep and sit without support. Neuropsychological development assessment was performed using the Children Neuropsychological and Behavioral Scale-Revision 2016 (CNBS-R2016) and the parent-rated Adaptive Behavior Assessment System II (ABAS-II) infant version at 3 years old. Her full-scale developmental quotient (DQ) of CNBS-R2016 was 115. The DQ in the five subscales involving gross motor, fine motor, adaptive behavior, language, personal-social of CNBS-R2016 were 120, 112, 112,120, and 112, respectively. The overall adaptive function score of ABAS-II was 106 (95% CI: 102–110, P66). The scores of social skills, conceptual skills, and practical skills in the three composite areas of adaptive function were 108 (95% CI 101–115, P70), 102 (95% CI 94–110, P55), and 106 (95% CI 99–113, P66), respectively. According to the neuropsychological development assessments, her intellectual development level was similar to that of children of the same age.

Her brain magnetic resonance imaging (MRI) scan at 4 months old revealed no parenchymal abnormality except for a left arachnoid cyst (20.1 mm × 11.5 mm × 10.1 mm). Electroencephalogram (EEG) at the same time showed partial spikes at left occipital-parietal and anterior temporal region during sleep. Possible hereditary metabolic diseases were screened from blood and urine samples by liquid chromatography tandem mass spectrometry (LC–MS/MS), and no abnormalities were found. No structural abnormalities of urinary, cardiac and digestive systems were found by color Doppler ultrasound when she was 4 months old. Ultrasonic diagnosis revealed dysplasia of her left hip joint at 1 month after birth, but returned to normal at 6 months of age.

#### Persistent mild anemia

**Figure 2:**
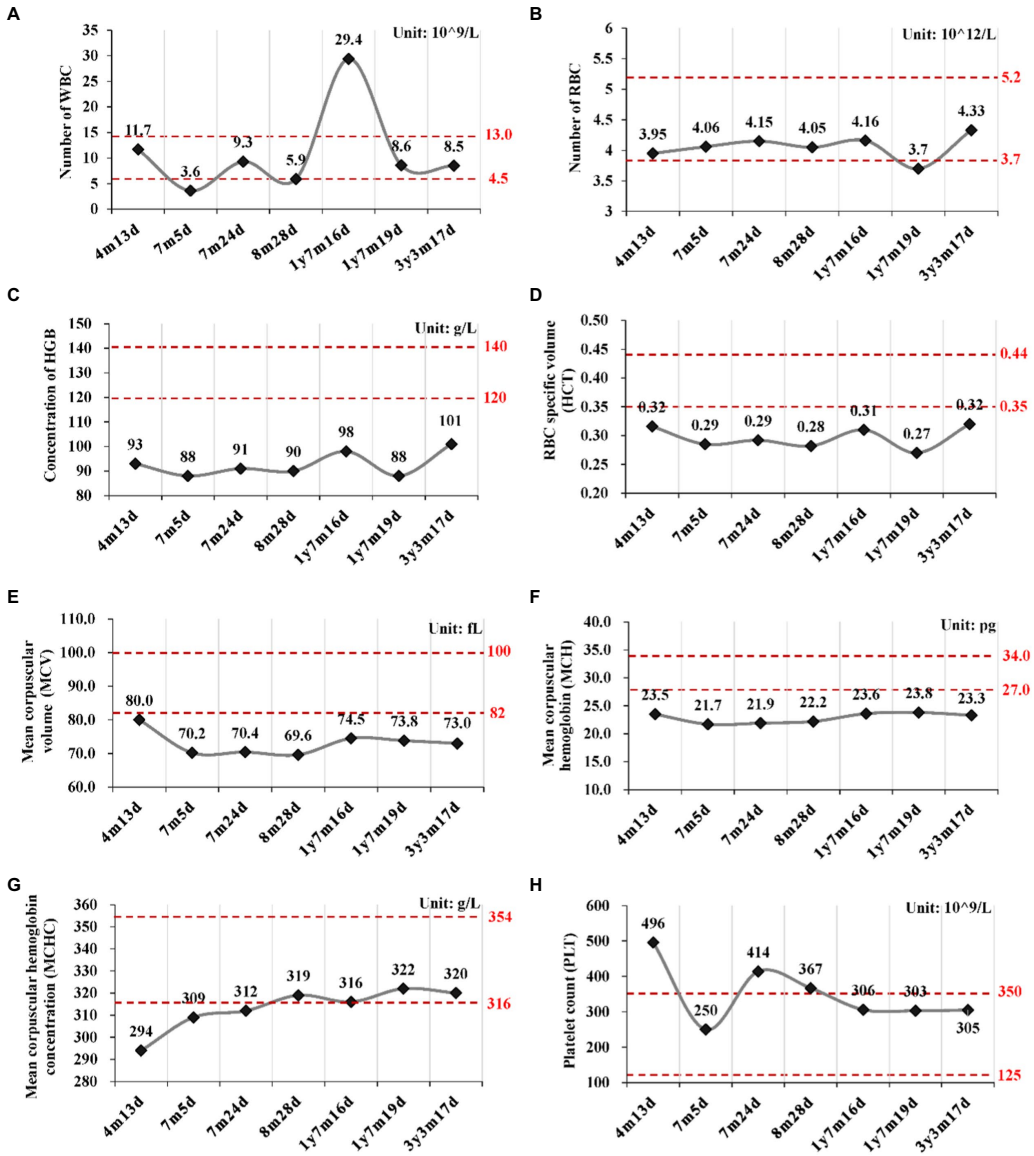
Routine blood tests at different time points. **(A)** Number of white blood cell (WBC); **(B)** Number of red blood cell; **(C)** Concentration of hemoglobin (HGB); **(D)** Hematocrit (HCT); **(E)** Mean corpuscular volume (MCV); **(F)** Mean corpuscular hemoglobin (MCH); **(G)** Mean corpuscular hemoglobin concentration (MCHC); **(H)** Platelet count (PLT). y, year; m, month; d, day.

Routine blood tests were performed seven times from 4 months to 3 years and 3 months after birth (Figure 2; Supplementary Table S2). No abnormality was found for the leukocyte and thrombocyte. Persistent mild anemia (HGB 88 ~ 98 g/L) with reduced mean corpuscular volume (MCV) and mean corpuscular hemoglobin (MCH) were observed, while the reticulocyte count was normal (36.3 × 10^9^/L, 0.9%). Peripheral blood smear showed normal morphology of leukocytes and platelets, and smaller volume of mature red blood cells with enlarged central light stained area. Bone marrow puncture was refused by her parent.

### Mutations in POLR3A and FANCA were identified by trio-WES

Karyotype analysis of G-banding chromosomes on peripheral blood mononuclear cells (PBMCs) detected no evident chromosomal abnormalities. Array-CGH analysis revealed no clinically significant microduplications or microdeletions. Trio-based whole exome sequencing (Trio-WES) was performed, and 53 rare variants were identified in the proband (Supplementary Table S3). All of the variants were inherited either from her father (parentally) or from her mother (maternally). Since the parent displayed no symptoms, genes with *de novo*, bi-allelic heterozygous or homozygous mutations were selected for subsequent analysis. Only one gene, *FANCA,* was remained in the list. Since homozygous or compound heterozygous mutations of *FANCA* gene resulted in the recessive Fanconi anemia of complementation group A (FANCA), this gene might be the unlying molecular factor for the anemia phenotype of the proband. However, no other genes were identified to be responsible for other clinical presentations.

**Figure 3:**
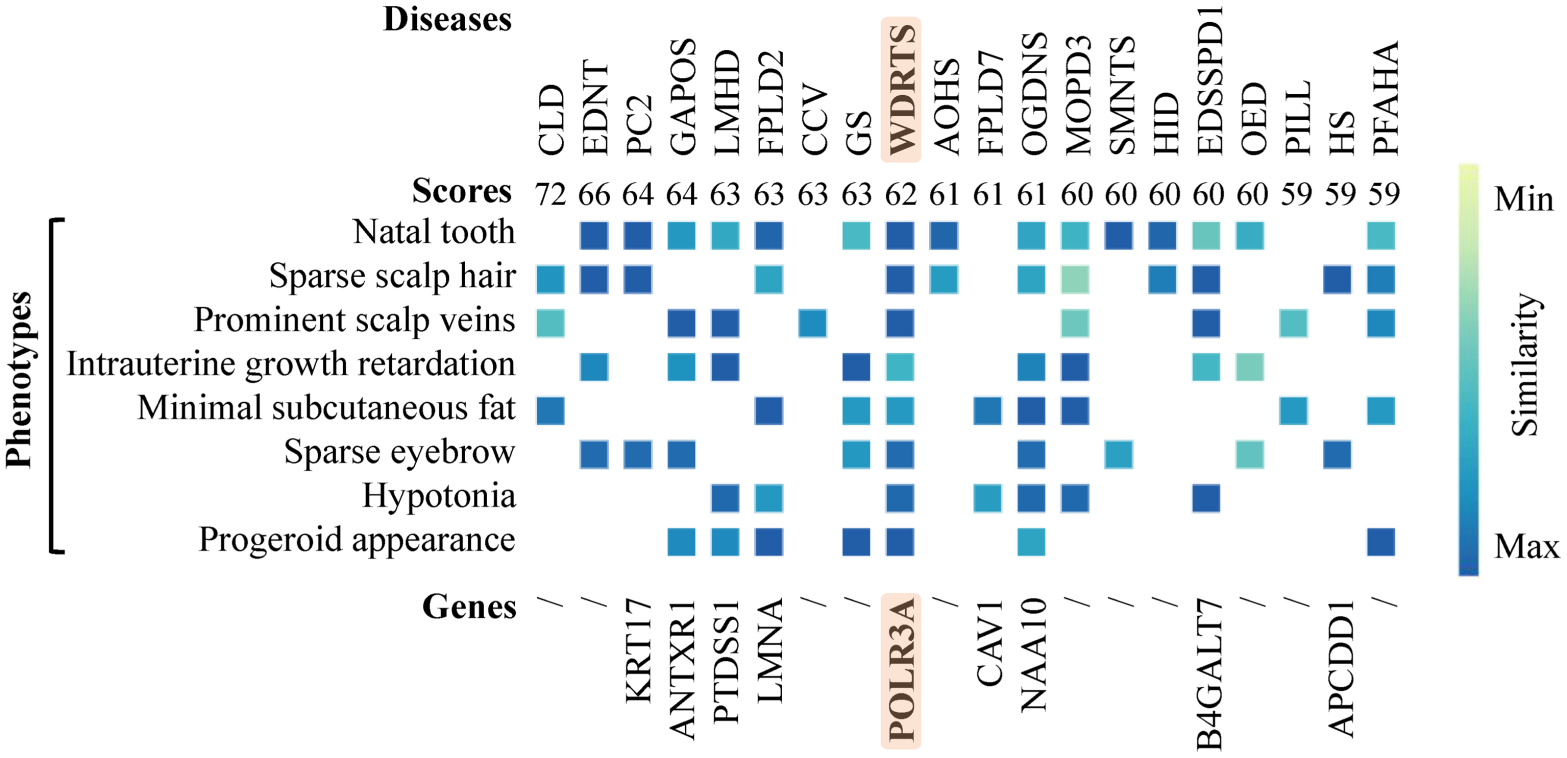
Phenotype profile search.

A Phenotype Profile Search was carried out at Human Phenotype Ontology (HPO) using eight key clinical features of the patient, such as progeroid facial appearance (HP:0005328), prominent scalp veins (HP:0001043), natal tooth (HP:0000695), intrauterine growth retardation (HP:0001511), and hypotonia (HP:0001252), sparse eyebrow (HP:0045075), sparse scalp hair (HP:0002209) and minimal (/thin) subcutaneous fat (HP:0003717). All eight input phenotypes were covered only in WDRTS, which was caused by mutations of *POLR3A* gene (Supplementary Table S4; Figure 3).

Only one rare mutation (c.3718G > A, p.Gly1240Ser) of *POLR3A* gene (NM_007055.4) was identified in the proband (Supplementary Table S3). Since WDRTS was an autosomal recessive (AR) disorder, the removed variants of *POLR3A* were revisited and one synonymous variant (c.3342C > T, p.Ser1114=) was retrieved (Table 1).

**Table 1:** Characterizations of mutations of POLR3A and FANCA.

Genes	Location (GRCh37/hg19)	HGVS annotation	SNP IP	Zygosity	ACMG classification	PROVEIN (score)	PolyPhen (score)	Minor allele frequencies (MAF)				
				P/F/M				1000Genome (*n* = 2,504)	TOPMED (*n* = 158,470)	ExAC (*n* = 60,706)	gnomAD (*n* = 76,156)	gnomAD_Exomes (*n* = 125,748)
POLR3A	10:79,743,765	c.3342C > T (p.Ser1114=)	rs183347762	Het/Het/WT	Likely Benign (PM2 + BP4 + BP6 + BP7)	Neutral 0.000	Benign 0.000	0.000200	0.000038	0.000036	0.000043	0.000036
POLR3A	10:79,741,953	c.3718G > A (p.Gly1240Ser)	rs1003620056	Het/WT/Het	Uncertain significance (PM2 + PP5)	Deleterious −5.480	Damaging 0.944	/	0.000016	0.000017	0.000007	0.000028
FANCA	16:89,839,791	c.1902 T > G (p.Asp634Glu)	rs187300458	Het/WT/Het	Likely Benign (PM2 + BP3 + BP4)	Neutral −0.420	Benign 0.034	0.000200	0.000008	/	0.000007	0.000006
FANCA	16:89,828,377	c.2832dup (p.Ala945CysfsTer6)	Novel	Het/Het/WT	Pathogenic (PVS1 + PM2 + PP5)	/	/	/	/	/	/	

HGVS, Human Genome Variation Society; P, patient; F, father; M, mother; Het, heterozygous; WT, wild type; ACMG, The American College of Medical Genetics and Genomics; PVS, Pathogenic, Very Strong; PM, Pathogenic, Moderate; PP, Pathogenic, Supporting; BP, Benign, Supporting; dup, duplication; Ter, termination.

### Molecular analysis of the bi-allelic mutations of *POLR3A* gene

**Figure 4:**
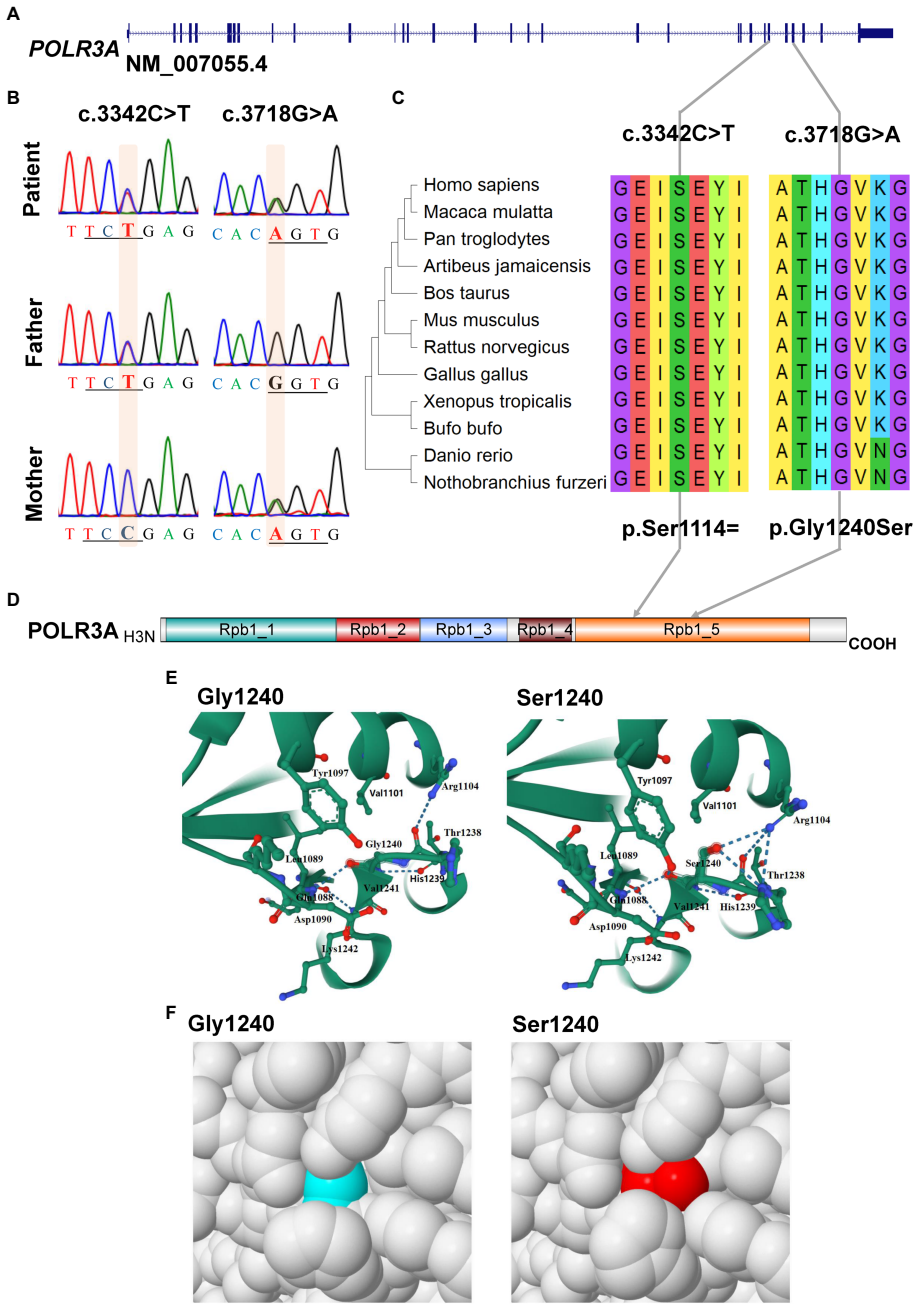
Characterization of mutations in *POLR3A* gene. **(A)** Diagram of genomic structure of *POLR3A*; **(B)** Sanger sequencing of the mutations; **(C)** Conservation analysis; **(D)** Diagram of protein structure of POLR3A; **(E)** 3-D structure of POLR3A; **(F)** Spacefill model.

For *POLR3A* gene, c.3718G > A (rs1003620056) in the exon 28 (28/31) was transmitted maternally (Figures 4A,B) and generated a missense mutation from Gly1240 to Ser1240 (NP_008986.2, p.Gly1240Ser) in the RNA_pol_Rpb1_5 domain of POLR3A (Figures 4C,D). The Gly1240 residual was highly conserved in different vertebrate species. This mutation was extremely rare in TOPMED (*n* = 158,470, MAF = 0.000016), ExAC (*n* = 60,706, MAF = 0.000017), gnomAD (*n* = 76,156, MAF = 0.000007), and gnomAD Exomes (*n* = 125,748, MAF = 0.000028). Besides, this mutation had been reported in two patients (4H-42 and 4H-67) with 4H leukodystrophy (Wolf et al., 2014). Functional predictions by Polyphen-2 and PROVEAN showed this variant to be “Damaging” (score = 0.995) or “Deleterious” (score = −5.480) to the proper function of POLR3A, respectively. According to the ACMG guidelines, this mutation was classified as “Uncertain Significance” (PM2 + PP5). The structural changes introduced by Gly1240Ser were analyzed by Missense3D according to the cryo-EM structure of human POLR3A protein (7d58, chain A, 2.9 Å resolution). It’s revealed that Gly1240 was originally buried in a bend curvature (RAS 0.0%), which could be disrupted by the substitution with the Ser1240 residue (RSA 1.5%). The Serine could form new hydrogen bonds with Arg1104, Thr1238, and Tyr1097 (Figure 4E), which changed the surfaces of the local structure (Figure 4F). The splicing potential of this mutation on the pre-mRNA of POLR3A was evaluated by SpliceAI and obtained negative index (score = 0.00; Supplementary Table S5). The retrieved variant, c.3342C > T (rs183347762) in exon 26 (26/31) of *POLR3A* gene, was inherited paternally. It was synonymous without changing the amino acid Serine at position 1,114 (p.Ser1114=) in the RNA_pol_Rpb1_5 domain of POLR3A protein, and conserved in different species (Figures 4C,D). This variant was extremely rare in the international projects for large human cohorts, such as 1,000 Genomes (*n* = 2,504, MAF = 0.000200), TOPMED (*n* = 158,470, MAF = 0.000038), ExAC (*n* = 60,706, MAF = 0.000036), gnomAD (*n* = 76,156, MAF = 0.000043), and gnomAD_exomes (*n* = 125,748, MAF = 0.000036). It was assessed to be “Benign” (score = 0.0000) or “Neutral” (score = 0.0000) by Polyphen-2 and PROVEAN, respectively. According to ACMG guidelines, this mutation was annotated as “Likely Benign” (PM2 + BP4 + BP6 + BP7). Although as a synonymous mutation, c.3342C > T was only six nucleotides away from the canonical splicing acceptor site (c.3337-1G, 10:79743771) of the intron 25 (IVS25). SpliceAI displayed negative result (score = 0.00, Supplementary Table S5). The prediction by SPIDEX indicated that this mutation might affect the proper pre-mRNA splicing of *POLR3A* to generate abnormal transcripts. According to the analysis by ESEFinder (version 3.0), the mutated allele (3,342 T) might generate a novel binding site (C**T**GAGTAT) for serine and arginine rich splicing factor 1 (SRSF1), which might affect the splicing pattern or efficiency of *POLR3A*.

### Molecular analysis of the bi-allelic mutations of *FANCA* gene

**Figure 5:**
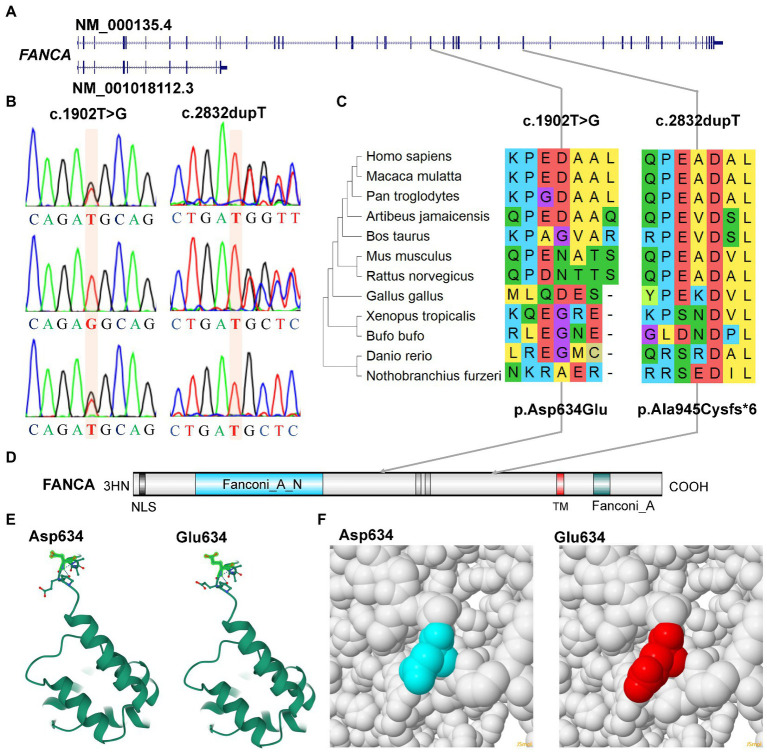
Characterization of mutations of *FANCA* gene. **(A)** Diagram of genomic structure of FANCA; **(B)** Sanger sequencing of the mutations; **(C)** Conservation of the mutations in different vertebrates; **(D)** Diagram of protein structure of FANCA; **(E)** 3-D structure of FANCA; **(F)** Space-filling model.

For the anemia phenotype, two rare mutations in the CDS of *FANCA* gene (NM_000135.4) were detected by trio-WES (Table 1). A single-nucleotide insertion (c.2832dup, 16:89828377) in the exon 29 (29/43) was identified to cause a frameshift to the FANCA protein (p.Ala945CysfsTer6, NP_000126.2; Figures 5A,B). This mutation had been reported in an 8-year-old female patient (Li et al., 2018). However, this mutation had not been identified in any of the four public human genome projects, 1000Genomes, TOPMED, ExAC, gnomAD and gnomAD_exomes databases. It was poorly conserved in different animal species (Figure 5C). The mutant transcript might be the target of nonsense-mediated mRNA decay (NMD) or encode a putative shortened protein lacking the transmembrane (TM) and C-terminal Fanconi_A domain (Figure 5D). According to ACMG guidelines, this mutation was annotated as “Pathogenic” (PVS1 + PM2 + PP5).

Another mutation in *FANCA*, c.1902 T > G (rs187300458) in exon 22 (22/43), was inherited maternally and changed the amino acid Aspartic acid (Asp., GAT) at position 634 to Glutamic acid (Glu, GAG; p.Asp634Glu; Figures 5A,B,D). This mutation was very rare in 1,000 Genome (*n* = 2,504, MAF = 0.000200), TOPMED (*n* = 158,470, MAF = 0.000008), gnomAD (*n* = 76,156, MAF = 0.000007), and GnomAD_exomes (*n* = 125,748, MAF = 0.000006). The conservation of this amino acid was very poor in different species (Figure 5C). This variant was predicted to be “Benign” (score = 0.255) or “Neutral” (score = −0.42) to the normal function of FANCA by Polyphen-2 or PROVEAN, respectively. According to ACMG guidelines, this mutation was annotated as “Likely Benign” (PM2 + BP3 + BP4). The structural changes introduced by Asp634Glu were analyzed by Missense3D according to the cryo-EM structure of human FANCA (7kzp, chain A, 3.1 Å resolution) and no structural damage was detected (Figures 5E,F). The c.1902 T > G was only two nucleotides away from the splicing acceptor site (c.1901-1G, 16:89839793, hg19) in intron 21 (IVS21) and might generate a novel putative splicing acceptor site (AT→AG). Negative impact (score = 0.03) of this missense on splicing of FANCA was identified by spliceAI (Supplementary Table S5). According to the prediction by ESEFinder (version 3.0), the mutant allele (1902G) might introduce novel binding sites for SRSF1 (CAGAGGC) and SRSF5 (ACAGAGG), and destroy a binding site for SRSF6 (TGCAGC). Although annotated to be benign, this mutation might affect the splicing pattern or efficiency of *FANCA* gene.

### Variants affected pre-mRNA splicing of *POLR3A* and *FANCA*

**Figure 6:**
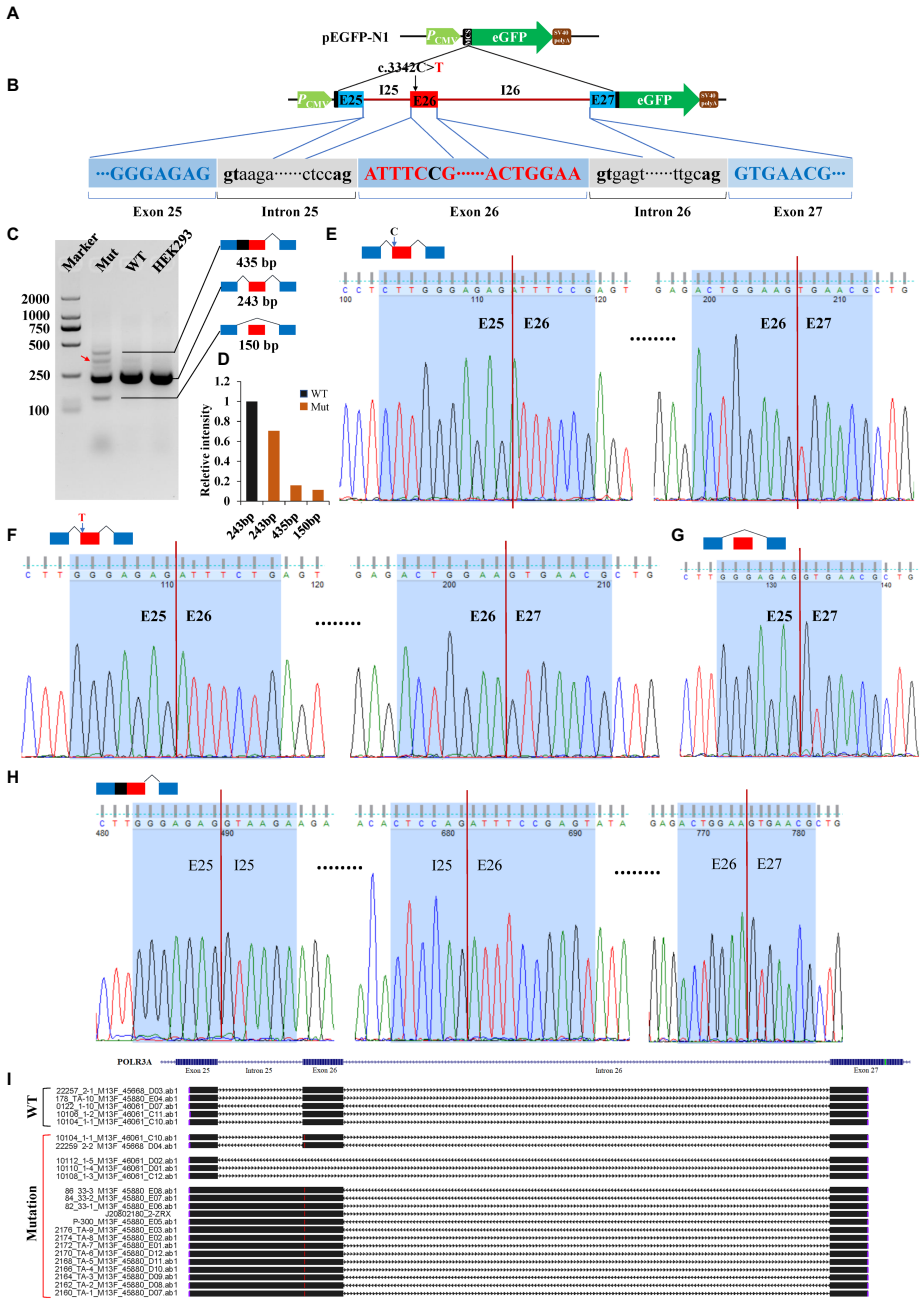
Minigene reporter assay for 3,342C > T in *POLR3A*. **(A)** Diagram of pEGFP-N1; **(B)** Positions of mutations; **(C)** Gel electrophoresis; **(D)** Intensity of bands; **(E)** Sanger sequencing for band 243 bp with 3,342C; **(F)** Sanger sequencing for band 243 bp with 3,342 T; **(G)** Sanger sequencing for band 150 bp; **(H)** Sanger sequencing for band 435 bp; **(I)** Alignment for sequenced PCR products. Red arrow indicates a non-specific band. WT, Wild type; Mut, Mutation; E, Exon; I, Intron.

Minigene reporter assay was carried out to verify the effects of c.3342C > T (*POLR3A*) and c.1902 T > G (*FANCA*) on pre-mRNA splicing. The genomic DNAs containing the selected mutations were cloned into the MCS of pEGFP-N1 and the mutations introduced by site-directed mutagenesis. As for c.3342C > T of *POLR3A*, the 1,646 bp genomic DNA spanning exon 25 to exon 27 (10:79,742,411-79,744,056) was cloned into the MCS (Figures 6A,B). Agarose electrophoresis revealed four different bands in the Mut samples, 435, 300, 243 bp and a short band (150 bp; Figure 6C). Sanger sequencing were carried out for the PCR products of these four bands and the band 300 bp was a non-specific product (pointed by the red arrow). The intensity of band 243 bp in Mut sample was about 70.60% of that in wild-type sample. The other two bands accounted for about 30% (Figure 6D). The band 243 bp was spliced with 3 exons (E25-E26-E27) and contained two types of isoforms, one wild type (Figure 6E) and one with 3,342 T (Figure 6F). The band 150 bp was produced by splicing between exon 25 and 27 to exclude the exon 26 (Figure 6G). In addition to the three consecutive exons, the band 435 bp retained the whole intron 25 (Figure 6H). After aligned all the Sanger-sequenced bands against *POLR3A* reference, three types of aberrant isoforms were revealed (Figure 6I).

**Figure 7:**
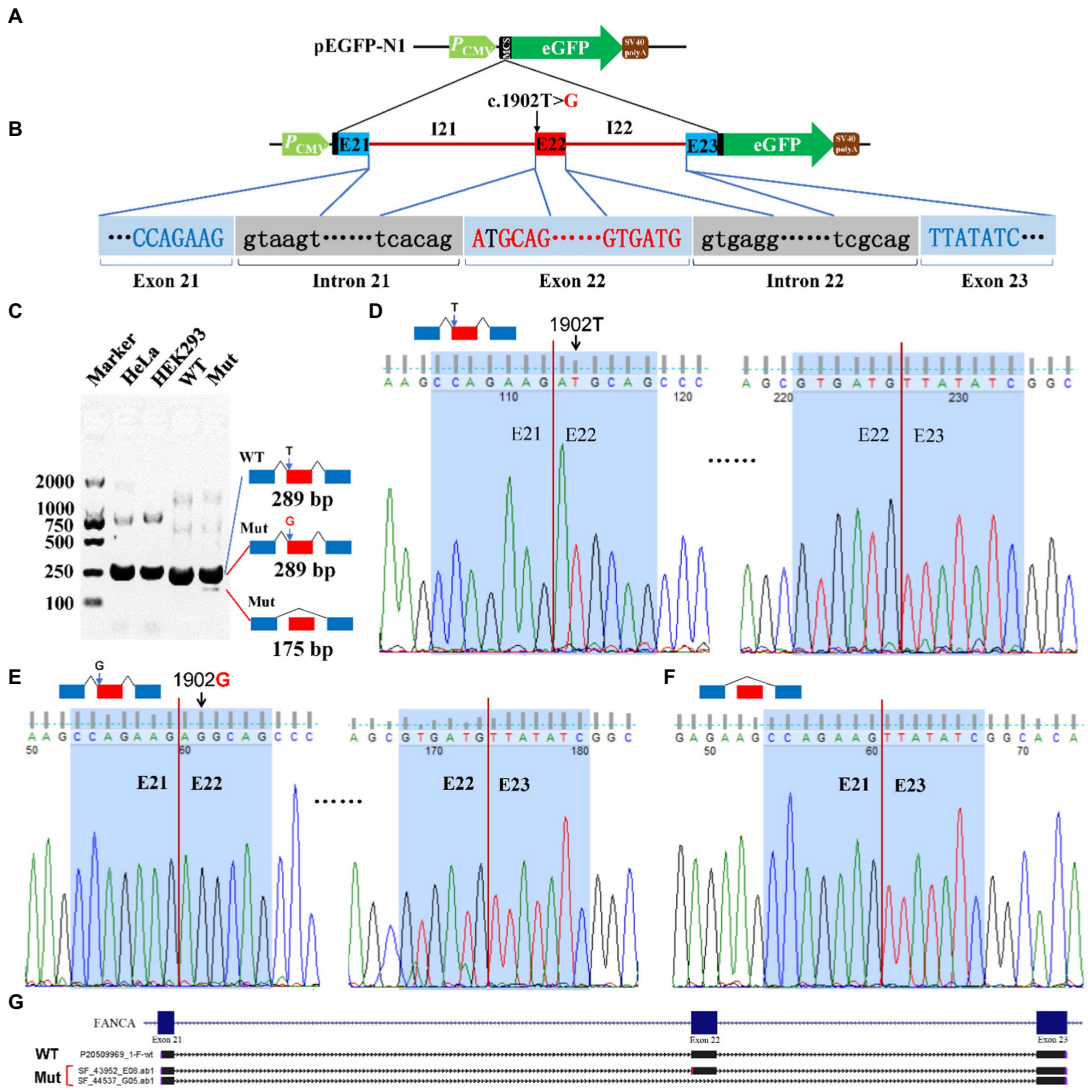
Minigene reporter assay for 1,902 T > G in *FANCA*. **(A)** Diagram of pEGFP-N1; **(B)** Positions of mutations; **(C)** Gel electrophoresis; **(D)** Sanger sequencing for the band 289 bp with 1,902 T; **(E)** Sanger sequencing for the band 289 bp with 1,902G; **(F)** Sanger sequencing for the band 175 bp; **(G)** Alignment for sequenced PCR products. WT, Wild type; Mut, Mutation; E, Exon; I, Intron.

For c.1902 T > G of *FANCA* gene, the 4,135 bp genomic DNA from exon 21 to exon 23 (16:89,838,089-89,842,223) was cloned into the MCS of pEGFP-N1 (Figures 7A,B). Agarose electrophoresis revealed a novel short band (175 bp), in addition to the long band (289 bp) in the Mut sample (Figure 7C). However, the staining of the short band was rather weak. Sanger sequencing were carried out for PCR products of the two bands. It is verified that the long band was produced by the consecutive splicing of three exons (E21-E22-E23; Figures 7D,E). The long band in the Mut sample contained the 1902G allele. The short band was spliced between exon 21 and exon 23, excluding exon 22 (Figures 7F,G).

### Anemia analysis

Alimentary anemia due to deficiencies of iron, vitamin B12, vitamin D and folic acid was not considered according to relevant biochemical tests (Supplementary Table S6). Regular supplementation of iron for 3 months was ineffective. Trio-WES found no mutations for thalassemia-related genes such as *HBA1* and *HBA2* for α-thalassemia and *HBB* for β-thalassemia (Supplementary Table S1). It had been reported that thalassemia could also be caused by long-fragment deletions, recombinations and mutations in locus control regions (LCRs) involving α- or β-globin genes, which could not be detected by WES. Therefore, a third-generation single molecule real-time (SMRT) sequencing for long-molecules containing thalassemia-related genes were carried out and no mutations were identified (Supplementary Table S7).

The mitomycin C (MMC)-induced chromosome stress assay was carried out for the peripheral blood samples from the patient and her mother, which was refused firmly by her father. After treated with different concentrations of MMC, 100 cells per sample were checked for chromosomal aberrations. However, no significant differences were observed between the two groups (Supplementary Figure S1).

**Figure 8:**
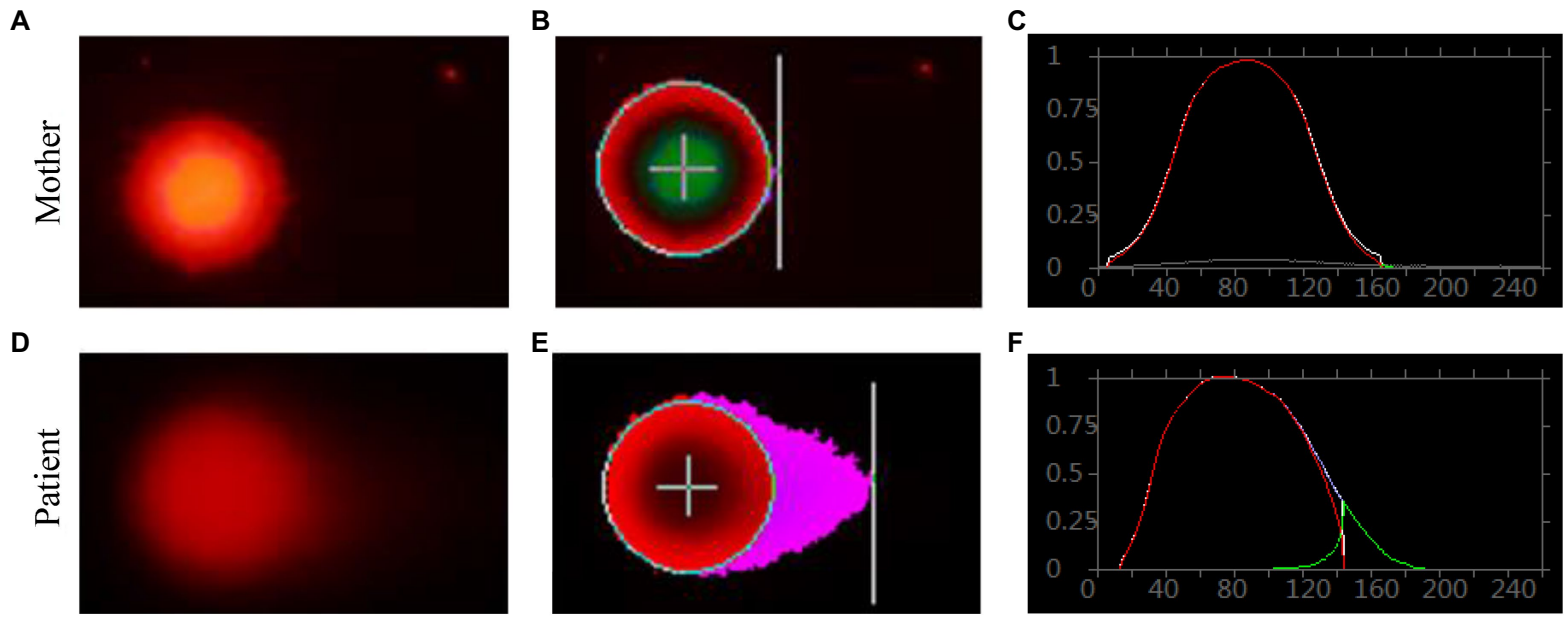
Single cell gel electrophoresis assay. Comet image of single lymphocyte from her mother **(A)**, and the patient **(D)**; CASP-analyzed comet image of single lymphocyte from her mother **(B)**, and the patient **(E)**; CASP analysis for her mother **(C)**, and the patient. **(F)** STRING network analysis involving POLR3A and FANCA.

Genomic DNA damages were measured through cell-based alkaline comet assay, which was performed by the single cell gel electrophoresis (SCGE). As shown in Figure 8, after exposure to alkaline lysis solutions, the control lymphocytes (mother) failed to show any comet-like fashion (Figures 8A–C). About 17% of the patient’s lymphocytes showed the appearance of an obscure “halo” around the nucleus (Figures 8D–F), but no apoptotic cells were identified. The comet tail length of the patient sample was longer than that of the control (Supplementary Table S8). TailDNA%, TM, and OTM of the patient were much higher than those of her mother (*p* < 0.001), indicating that the level of DNA damage in the patient who carried FANCA mutations was higher than those in the control.

**Figure 9:**
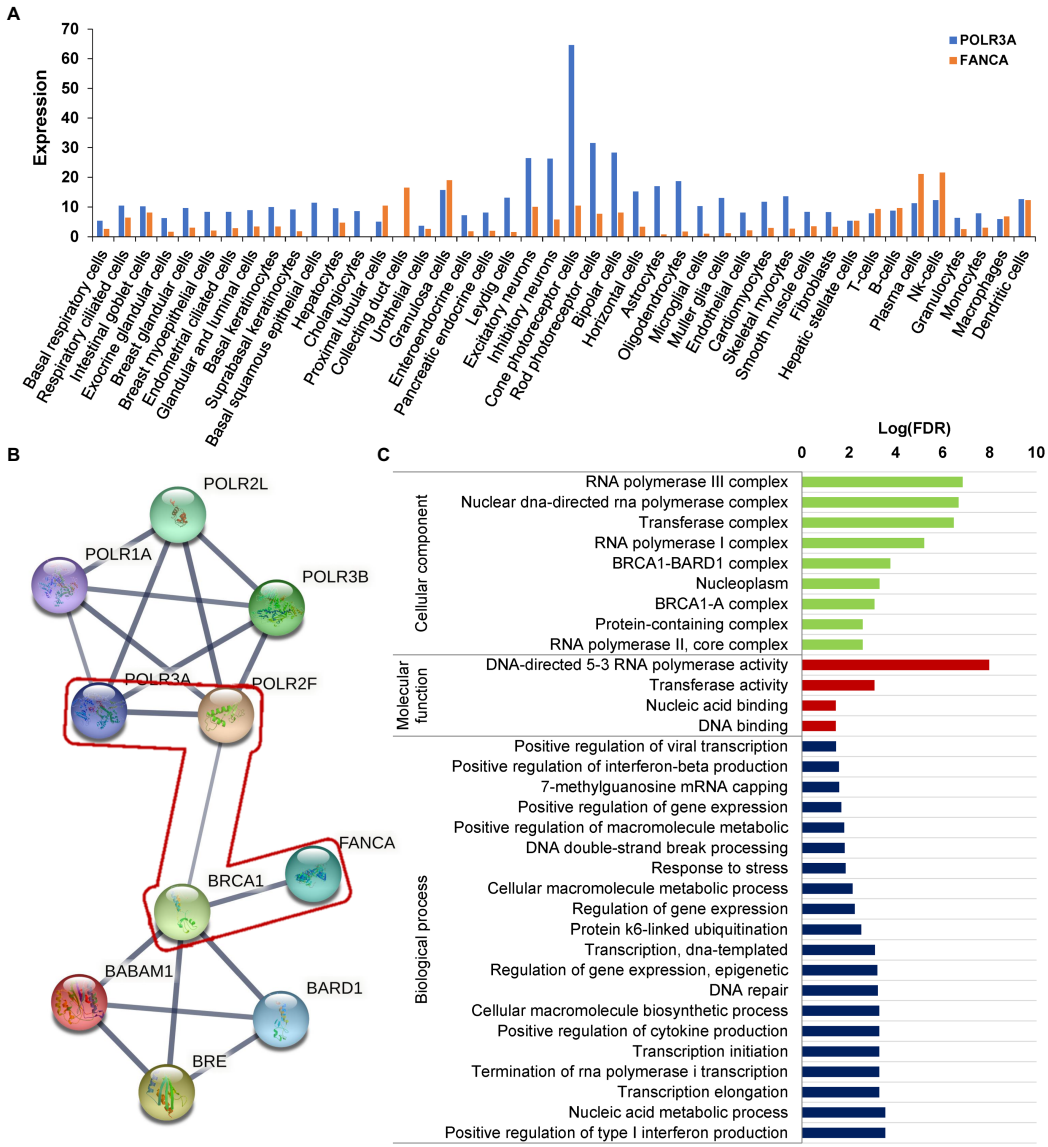
Protein network analysis of POLR3A and FANCA. **(A)** Expression in different human tissues; **(B)** STRINGed Network of POLR3A and FANCA; **(C)** GO analysis of the members of the network.

RNA polymerase III was essential for the homologous recombination-dependent repair of DNA double-strand breaks (DSBs) (Liu et al., 2021) and FANCA was involved in inter-strand cross-link repair (Knipscheer et al., 2009). These two genes might act synergistically in this patient. According to the gene expression data of 79 human tissues, *POLR3A* and *FANCA* were co-expressed in many different tissues (Figure 9A). Ten proteins involving POLR3A and FANCA could form a stringed network (PPI enrichment *value of p* = 5.92E-10). The network showed that POLR3A could interact directly with POLR3B, POLR1A, POLR2F, and POLR2L to form a multi-subunit RNA polymerase complex possessing the DNA-directed 5′-3′ RNA polymerase activity (FDR = 4.63E-08; Figure 9B). FANCA could bind directly with BRCA1, which was an important component of the BRCA1-A complex (BRCA1, BARD1, BABAM1, and BRE; FDR = 1.92E-08). Interestingly, through the nodes of BRCA1 and POLR2F, FANCA could be stringed with POLR3A. Although all of the 10 proteins were involved in the nucleic acid metabolic process (FDR = 3.84E-06; Figure 9C), the synergistic function of FANCA on RNA polymerization III or *vise verse* was remained for further exploration.

## Discussion

The gene *POLR3A* is located on chromosome 10q22.3, with 31 exons to encode a protein of 1,391 amino acid having a molecular mass of 154.7 kilodaltons. POLR3A is the largest catalytic subunit of the DNA-directed RNA polymerase III complex, which transcribes genes responsible for many small non-coding RNAs (ncRNAs), such as ribosomal 5S RNA, tRNAs, U6 small nuclear RNA, RNA components of mitochondrial RNA processing endoribonuclease (RMRP), ribonuclease P RNA component H1 (RPPH1), Ro60-associated RNA Y1 (RNY1), RNA component of signal recognition particle 7SL1 (RN7SL1) and RNA component of 7SK nuclear ribonucleoprotein (RN7SK). Some of these ncRNAs, such as RN7SL1 and RN7SK, regulate the activity of DNA-dependent RNA polymerase II, hence POLR3A mutations can also affect expression levels of polymerase II-transcribed genes (Azmanov et al., 2016; Flynn et al., 2016; Egloff et al., 2018). POLR3A also acts as a sensor to detect foreign viral DNAs and triggers an innate immune response (Ablasser et al., 2009). Recently, it has been reported that RNA polymerase III is an essential factor in the homologous recombination-dependent repair of DNA double-strand breaks (DSBs; Liu et al., 2021; Liu and Kong, 2021). Inhibition of POLR3A (also called Rpc1) could lead to the loss of genes in the DSB regions (Liu et al., 2021). Since POLR3A is ubiquitously expressed, the disability of this gene might be fatal to the prenatal and postnatal development of many systems.

It had been reported that pathologic homozygous or bi-allelic heterozygous mutations in *POLR3A* could cause the occurrence of Wiedemann-Rautenstrauch syndrome (WDRTS; Paolacci et al., 2018) or Hypomyelinating leukodystrophy 7 (HLD7, OMIM# 607694) (Bernard et al., 2011) under an autosomal recessive (AR) mode of inheritance. WDRTS was one of the rare disorders having neonatal progeroid phenotype. The others included fontaine progeroid syndrome (FPS, OMIM#612289) (Writzl et al., 2017), autosomal recessive cutis laxa type IIIA (ARCL3A, OMIM#219150), and some forms of Marfan syndrome (MFS; Graul-Neumann et al., 2010; Takenouchi et al., 2013; Garg and Xing, 2014; Jacquinet et al., 2014). These syndromes had some characteristics similar to WDRTS. Besides, the clinical phenotypes of WDRTS were highly variable involving many systems. In addition, a variant of WDRTS was reported to have some atypical WDRST features (such as no lipodystrophy, no natal teeth and no sparse scalp hair), which was caused by a homozygous mutation in *POLR3GL* (c.358C > T, p.Arg120Ter) (Beauregard-Lacroix et al., 2020). These factors together made it difficult to accurately discriminate the WDRTS from other disorders having similar phenotypes. After checking clinical presentations of the above-mentioned syndromes, patients affected with WDRTS had neonatal tooth or teeth abnormalities, those with other neonatal progeroid phenotypes did not. It seemed that neonatal tooth might be an essential marker to discriminate WDRTS from other disorders having progeroid facial features.

For our patient, she carried bi-allelic mutations in the CDS of *POLR3A* (c.3342C > T, p.Ser1114 = and c.3718G > A, p.Gly1240Ser). Except for c.3718G > A (p.Gly1240Ser) which could affect the structural conformation of POLR3A protein, the synonymous variant (c.3342C > T, p.Ser1114=) could lead to three types of abnormally spliced isoforms. The isoform 243 bp was consecutively spliced with three exons and carried the mutant allele. The isoform 435 bp was generated by the retention of intron 25, plus the three consecutive exons. After analyzed by Open Reading Frame Finder (ORF Finder), there was a premature stop codon in the intron 25 and might be translated into an aberrant protein (p.Glu1112GlufsTer7) or degraded by the nonsense-mediated mRNA decay (NMD; Lykke-Andersen and Jensen, 2015; Karousis and Muhlemann, 2019). As for the short isoform 150 bp, it only contained two exons (exon 25 and 27). Since the length of exon 26 was 93 base pairs (a multiple of three), the CDS of *POLR3A* should be left intact but missing 31 amino acids (aa1113-1,143) in the RNA_pol_Rpb1_5 domain (aa841-1,315). There were 4 missense mutations in the excluded exon 26 which were recruited in the NCBI ClinVar database, c.3350 T > C (p.Ile1117Thr), c.3388G > A (p.Val1130Ile), c.3392A > G (p.Lys1131Arg) and c.3407G > A (p.Arg1136Gln). These mutations were identified in patients with WDRTS or HLD7. In addition, c.3392A > G (p.Lys1131Arg) had been reported in a Caucasian WDRTS patient by targeted parallel sequencing (Paolacci et al., 2018). This indicated that the excluded region might be important for the function of POLR3A. Since also having core clinical phenotypes of WDRTS (Paolacci et al., 2017), the female proband was finally diagnosed as WDRTS caused by bi-allelic mutations in *POLR3A*. According to the mini-gene reporter assay, there were about 70% full-length wild-type and synonymous-containing transcripts. This indicated that a pathogenic hierarchy might be related to the two mutations. The missense mutation, c.3718G > A (p.Gly1240Ser), was the major contributor to the clinical presentations of our patient, with c.3342C > T (p.Ser1114=) as the minor one.

Except for missense, nonsense, frameshifting, and mutations disrupting canonical splicing sites, there were 10 intronic mutations to affect pre-mRNA splicing of *POLR3A* (Hiraide et al., 2020), such as c.645 + 312C > T (Hiraide et al., 2020), c.1048 + 5G > T (Minnerop et al., 2017), c.1770 + 5G > C (Yan et al., 2021), c.1771-6C > G (Rydning et al., 2019; Wu et al., 2019), c.1771-7C > G (Minnerop et al., 2017), c.1909 + 22G > A (Minnerop et al., 2017; Morales-Rosado et al., 2020), c.1909 + 18G > A (Lessel et al., 2018), c.2003 + 18G > A (Bernard et al., 2011), c.3337-5 T > A (Lessel et al., 2018; Wambach et al., 2018), c.3337-11 T > C (Wambach et al., 2018). Among them, c.1909 + 22G > A was the most commonly reported mutations. However, synonymous variants affecting the pre-mRNA splicing of POLR3A were rarely reported. Till now, only one homozygous synonymous (c.3336G > A, p.Glu1112=) has been reported to generate two types of abnormal splicing isoforms, one with the retention of intron 25, and another with the exclusion of exon 25 (Lessel et al., 2022). Our patient was the second report of a synonymous variant to affect the pre-mRNA splicing of POLR3A.

**Figure 10:**
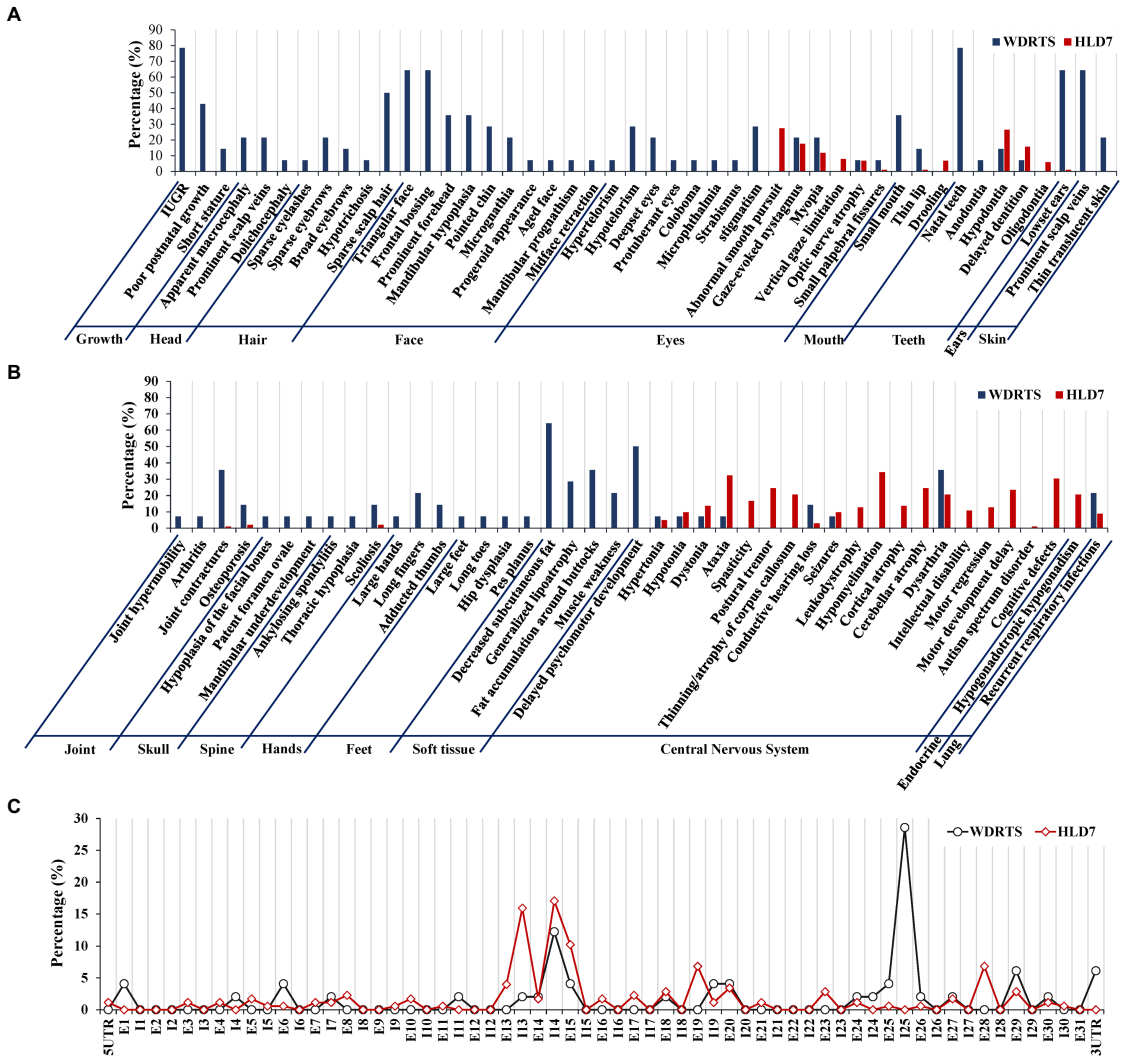
Phenotype and genotype analysis for WDRTS and HLD7. **(A,B)** Phenotypes in different organs; **(C)** Distributions of mutations in exons and introns of POLR3A. E, exon; I, intron. UTR, untranslated region.

In order to have a comprehensive view of the phenotypes of WDRTS and HLD7, a literature review was made. Features of craniofacial dysmorphism and soft tissues were exclusively confined to WDRTS (Figures 10A,B). However, the majority of the abnormal phenotypes in central nervous system were mainly found in patients suffering from HLD7 (Figure 10B). The reported mutations of POLR3A in WDRTS and HLD7 were compiled and arranged according to their genomic position. To our surprise, there were a few different “hot spots” between WDRTS and HLD7. Mutations in intron 13, exon 19 and exon 28 were almost exclusively related to HLD7. For WDRTS, most of the mutations were distributed in exon 1, exon 6, intron 25 and 3’-UTR, with intron 25 as the highest (Figure 10C). For our patient, the splicing-altering mutation c.3342C > T was located at the junction between exon 25 and intron 25. It seemed that the aberrant pre-mRNA splicing at intron 25 might be correlated with the occurrence of WDRTS.

Efforts to establish animal models with Polr3a mutation had been tried in mice, but had not been successful. It had been reported that double knockout (KO) of polr3a in mice was embryonically lethal (Choquet et al., 2019). Furthermore, no neurological or developmental abnormalities were identified in mice with whole-body homozygous knock-in (KI/KI) or heterozygous KI/KO of the pathogenic c.2015G > A (p.Gly672Glu) mutation of polr3a (Choquet et al., 2017). For RNA polymerase III (POLR3) in animals, it composed of 17 subunits to form a catalytic core, the stalk domain and Pol III-specific subcomplexes (Vannini and Cramer, 2012; Girbig et al., 2021). Till now, only six of 17 subunits (35.29%) were reported to be the causal for a spectrum of rarely inherited disorders. Mutations in *POLR3A* were responsible for WDRTS or HLD7 (Bernard et al., 2011; Wambach et al., 2018), *POLR3B* for HLD8 (OMIM#614381; Saitsu et al., 2011), *POLR1C* for HLD11 (OMIM#616494) (Thiffault et al., 2015), *POLR3K* for HLD21 (OMIM#619310) (Dorboz et al., 2018), *POLR3GL* for short stature, oligodontia, dysmorphic facies, and motor delay (SOFM, OMIM#619234; Terhal et al., 2020) and *POLR3H* for primary ovarian insufficiency (POI; Franca et al., 2019). Since only 35.29% of the members of POLR3 could be related to inheritable disorders, there might be a functional redundancy among other subunits.

Inferred from the time-coursed routine blood testing, the patient had a moderate level of anemia. The anemia belonged to small cell hypochromic anemia, similar to iron deficiency anemia or thalassemia. However, the concentrations of serum ferritin, vitamin B12, folic acid and vitamin D were within a normal range, indicating that the anemia might be caused by other unknown reasons. Through trio-WES, two mutations were identified in the CDS of *FANCA*, a causal gene for Fanconi anemia of complementation group A (FANCA, OMIM#227650). No mutations were identified in genes responsible for thalassemia by trio-WES and the third-generation SMRT sequencing. For *FANCA*, the pathogenic insertion (c.2832dup) in exon 29 introduced a premature termination codon (PTC), which caused a frameshift of the FANCA protein (p.Ala945CysfsTer6) or rendered the resultant transcripts to be rapidly degraded by NMD. Another missense mutation (c.1902 T > G, p.Asp634Glu) was predicted to be benign. After carefully analyzing the genomic sequence containing c.1902 T > G, it was only two nucleotides away from the canonical splicing acceptor site (SA1, c.1901-1_1901–2, AG) in intron 21. The mutation might introduce a potential splicing acceptor site (SA2, c.1902_1903, AG) juxtaposed with SA1. Minigene reporter assay identified two types of aberrant isoforms. One carried the 1902G and translated into a full-length FANCA protein with Glu634. The other isoform was produced by splicing between exon 21 and 23 to exclude exon 22, but at a very low level. Since the length of exon 22 was 114 base pairs (a multiple of three), the CDS of *FANCA* should be left intact but missing 38 amino acids (aa634-672). However, the function of this region was not clear. There were seven pathogenic mutations in this excluded region recruited in the ClinVar database, namely, c.1912G > T (p.Gly638Ter), c.1944del (p.Glu648AspfsTer13; Levran et al., 1997), c.1951G > T (p.Gly651Ter), c.1979 T > C (p.Leu660Pro; Ameziane et al., 2008), c.1981A > T (p.Arg661Ter), c.2001dup (p.Ser668GlnfsTer4; Moghrabi et al., 2009), and c.2005C > T (p.Gln669Ter). This indicated that the excluded region was important for the function of FANCA protein. According to the mini-gene reporter assay, there were more than 80% full-length wild-type and missense-containing transcripts. A pathogenic hierarchy might be related to the two mutations. The frameshift mutation, c.2832dup (p.Ala945CysfsTer6), was the major contributor to the clinical presentations of our patient, with the missense c.1902 T > G (p.Asp634Glu) as the minor one. This indicated that there might be a delicate balance between wild-type and mutated transcripts to prevent the occurrence of macroscopic clinical phenotypes.

In order to verify the phenotype of Fanconi’s anemia (FA), mitomycin C-induced chromosome stress (MMC) assay and single cell gel electrophoresis (SCGE) assay were performed for blood samples from the patient and her mother. MMC assay detected no significant chromosome aberrations. For SCGE assay, good-shaped comets were observed in the patient’s sample, indicating that the genomic DNA was seriously damaged. This implied that the patient might suffer from a mild type of FA without obvious classical phenotypes.

To our knowledge, this was the first report of a patient with a rare Wiedemann-Rautenstrauch syndrome (WDRTS) complicated with another recessive disorder, Fanconi anemia of complementation group A (FANCA). It had been reported that both POLR3A and FANCA were involved in the homologous recombination-dependent repair of DNA double-strand breaks (DSBs) (Liu et al., 2021; Liu and Kong, 2021) and inter-strand DNA cross-link repair (Howard et al., 2015; Benitez et al., 2018) to maintain the chromosome stability. A network analysis showed that POLR3A could be STRINGed with FANCA *via* two nodes of BRCA1 and POLR2F (Krum et al., 2003; Lane, 2004). It is implied that both proteins might act synergistically to contribute to the complexity of clinical phenotypes. This should be verified by further cellular and model animal experiments.

## Conclusion

Generally, a WDRTS patient was identified to have rare bi-allelic compound mutations in *POLR3A*, one damaging missense and one synonymous. The synonymous mutation could affect the pre-mRNA splicing of *POLR3A* and should be pathogenic. It generated about 30% of aberrantly splicing transcripts. As for the anemia phenotype, the predicted benign missense mutation 1902 T > G could generate a small proportion of abnormally spliced isoform of FANCA. The expressed ratio between the aberrant and wild type isoforms might be correlated to the severity of the disease. Even patients carrying same splicing-altering mutations presented different phenotypes, other unidentified regulatory polymorphisms might be the modifying factors for the different penetration. Since the detrimental level of mutations vary greatly, different combinations of these mutations might be one of the underlying mechanisms for the varied clinical phenotype penetrance and prognosis. It might be very useful for clinical genetic consultors to have a comprehensive analysis for the relationship between genetic factors and clinical features.

## Data availability statement

The datasets presented in this study can be found in online repositories. The names of the repository/repositories and accession number(s) can be found in the article/Supplementary material.

## Ethics statement

The studies involving human participants were reviewed and approved by Ethical Committee of the Shenzhen Baoan Women’s and Children’s Hospital. Written informed consent to participate in this study was provided by the participants’ legal guardian/next of kin.

## Author contributions

GL and QP designed the study. GL and YZ analyzed gnomic data. QP, BX, JD, ZX, and BD provided phenotype information. QP and BD performed followup inquiries. LW, WD, DL, and JW performed the minigene reporter assay, SCGE assay, and MMC assay. GL wrote the manuscript. GL, XH, and QP revised the manuscript. All authors contributed to the article and approved the submitted version.

## Funding

This work was supported by fundings from the Science and Technology Research and Development Foundation of Shenzhen (JCYJ20180305164359668), Natural Science Foundation of Sichuan (2022NSFSC0714), Key Research and Development Project of Deyang science and Technology Bureau (2021SZ003 and 2020SZZ085), and Special Fund for Incubation Projects of Deyang People’s Hospital (FHG202004).

## Conflict of interest

The authors declare that the research was conducted in the absence of any commercial or financial relationships that could be construed as a potential conflict of interest.

## Publisher’s note

All claims expressed in this article are solely those of the authors and do not necessarily represent those of their affiliated organizations, or those of the publisher, the editors and the reviewers. Any product that may be evaluated in this article, or claim that may be made by its manufacturer, is not guaranteed or endorsed by the publisher.

## Footnotes

^1^ https://github.com/Illumina/SpliceAI

^2^ https://www.pdbus.org/3d-view

^3^ http://geneontology.org/
