# Supplementary material for: A synonymous variant contributes to a rare Wiedemann-Rautenstrauch syndrome complicated with mild anemia via affecting pre-mRNA splicing
Source: Front Mol Neurosci. 2022 Oct 28;15:1026530. doi: 10.3389/fnmol.2022.1026530 (PMC9649808; doi:10.3389/fnmol.2022.1026530)
Supplement: Supplementary file 1 [file Data_Sheet_1.docx]

# Supplementary Data of WDRTS

### Thalassemia genetic variants based on third-generation sequencing

The third-generation sequencing for thalassemia-related genes was performed as previously described [25-27]. Briefly, genomic DNA was amplified by multiplex long PCR with primers targeting known mutations, structual variations, and recombinations for the HBA1, HBA2 and HBB genes. For the amplified PCR products, barcoded adaptors were ligated to construct single molecule real-time (SMRT) sequencing libraries by T4 DNA ligase. The ligated libraries were quantified and equal volumes of each samples pooled together. The pooled libraries were converted to SMRT libraries by annealing sequencing primers to the single-stranded loop region of the SMRT template, and then the primer-annealed templates bound to Sequel Polymerase 3.0 using the Sequel Binding and Internal Ctrl Kit 3.0 (Pacific Biosciences). SMRT cells were loaded with DNA-polymerase complexes and sequenced on the Sequel II platform (Pacific Biosciences).

A Bam file output from the PacBio Sequel System was used for subsequent analysis. Raw reads were demultiplexed, and debarcoded by using lima in the Pbbioconda package (Pacific Biosciences) to generate circular consensus sequencing (CCS) reads using the CCS software. Finally, the generated CCS reads were aligned to human reference genome (hg19) by using CoNvex Gap-cost alignMents for Long Reads (NGMLR, version 0.2.623). For identification of single-nucleotide variations (SNVs), small indels, and structural variations, the CCS reads were re-aligned to the reference genome using BLASR and variants called using FreeBayes (version 1.3.4).

### Mitomycin C-induced chromosome stress assay

The mitomycin C-induced chromosome stress assay was carried out as previously described [28, 29]. Briefly, 0.5 mL peripheral blood in sodium heparin was mixed with 5 mL RPMI1640 medium (20% inactivated fetal bovine serum and 50 μg/mL PHA) and incubated at 37°C in a 5% CO_2_ atmosphere. After 44 hours incubation, 40 ng/mL mitomycin C (MMC) was added into the mixture and incubated for another 3 hours. Cytochalasin B (6 μg/mL) was added and incubated for 28 hours. The incubated cells were collected and suspended in a mild hypotonic solution (75 mM KCl) and fixed with methanol:acetic acid (3:1). Then, the cells were dropped onto precleaned slides and stained with 4% Giemsa. A total of 100 metaphase cells per subject were analyzed and scored for the number and type of chromosome aberrations. Patients were regarded as having Fanconi anemia when more than 90% of the cells showed increased chromosomal abberations.

## Supplementary figures

Figure S1. MMC assay for blood samples from the patient and her mother


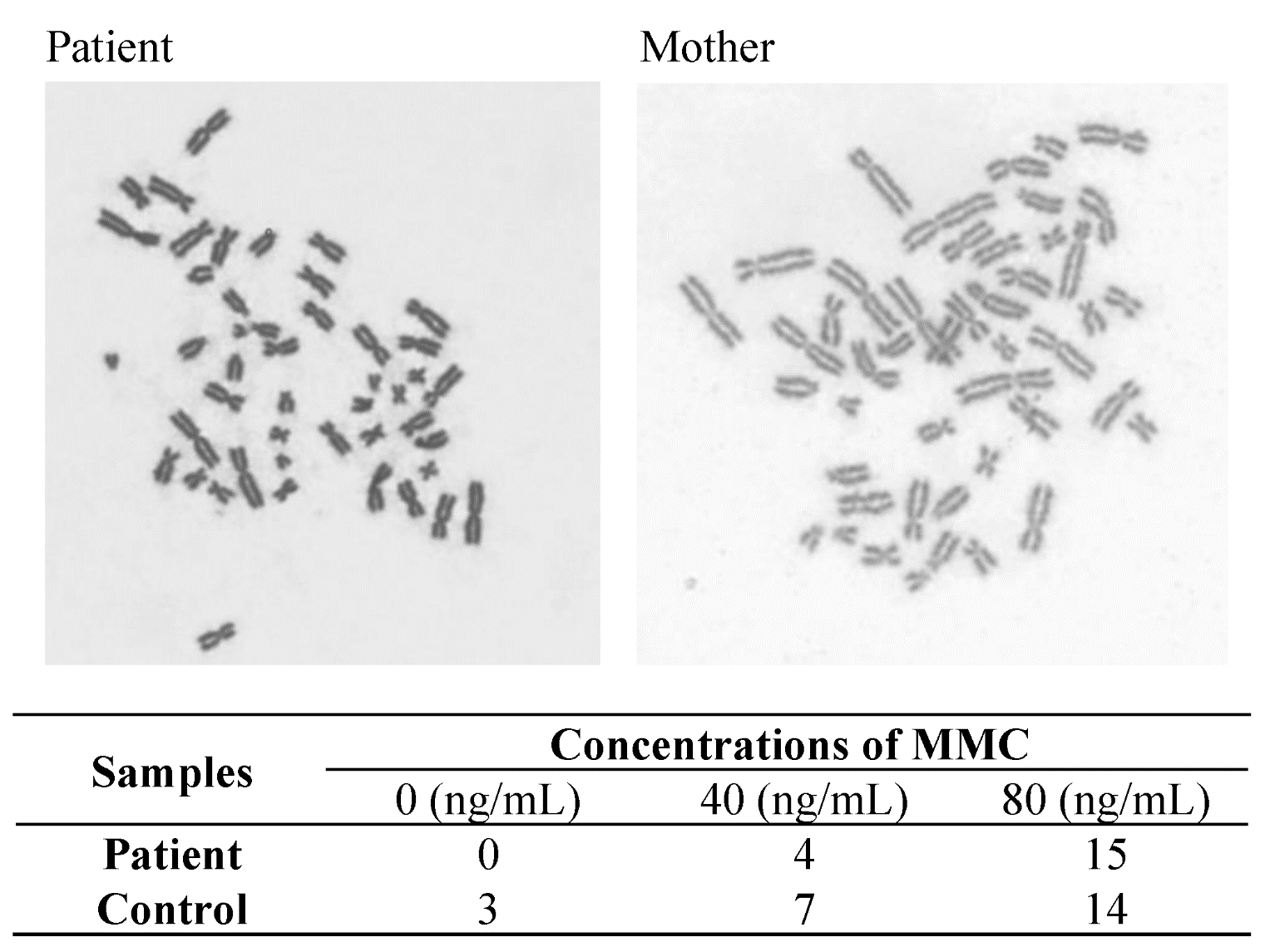


## Supplementary tables

### Table S1. Primers for the POLR3A and FANCA minigene assays

| **Genes** | **Direction** | **Sequence** | **Length**  **(bp)** | **Product size**  **(bp)** |
| --- | --- | --- | --- | --- |
| POLR3A | Forward | GGATGACGACGCGGATTATGCT | 22 | 243 |
| POLR3A | Reverse | ACAGCCTCACCATGAACAGCC | 21 |  |
| FANCA | Forward | ATGGTCTGTACTCCACCTACTGCC | 24 | 289 |
| FANCA | Reverse | AGTCTCGGCGTGTTGATGCTG | 21 |  |

### Table S2. Routine blood tests for the proband

| **Items** | **Results (S.D.)** | **Units** | **Reference range** |
| --- | --- | --- | --- |
| White blood cell count (WBC) | 7.820 (3.136) | 10^9/L | 4.5-13 |
| Neutrophil ratio (NEUT%) | 42.240 (16.310) |  | 42.9-74.3 |
| Lymphocyte ratio (LYMPH%) | 43.56 0(17.055) |  | 18.3-45.7 |
| Monocyte ratio (MONO%) | 9.360 (5.644) |  | 4.2-11.8 |
| Eosinophil ratio (EO%) | 2.620 (1.003) |  | 0.2-5.3 |
| Basophil ratio (BASO%) | 0.220 (0.084) |  | 0.1-1.0 |
| **Red blood cell count (RBC)** | **4.012 (0.171)** | 10^12/L | 3.7-5.2 |
| **Hemoglobin (HGB)** | **91.333 (3.777)** | g/L | 120-140 |
| **Hematocrit (HCT)** | **29.250 (1.750)** |  | 35-44 |
| **Mean corpuscular volume (MCV)** | **73.083 (3.950)** | fL | 82-100 |
| **Mean corpuscular hemoglobin (MCH)** | **22.783 (0.950)** | pg | 27-34 |
| **Mean corpuscular hemoglobin concentration (MCHC)** | **312.000 (9.980)** | g/L | 316-354 |
| Platelet count (PLT) | 356.000 (89.028) | 10^9/L | 125-350 |
| Thrombocytocrit (PCT) | 0.433 (0.162) |  | 0.18-0.39 |
| Mean platelet volume (MPV) | 11.600 (0.917) | fL | 9.1-11.9 |

### Table S3. Identified mutations in the trio by whole exome sequencing

| **No.** | **Genes** | **Location**  **(hg19)** | **Transcript** | **Exons** | **HGVS** | **dbSNP ID** | **Zygosity**  **(P/F/M)** | **MAF** | **Phenotype** | **OMIM** | **MOI** |
| --- | --- | --- | --- | --- | --- | --- | --- | --- | --- | --- | --- |
| **1** | **FANCA** | **16:89828377** | **NM_000135** | **29** | **c.2832dup**  **(p.Ala945CysfsTer6)** | **Novel** | **Het/Het/WT** | **/** | **FANCA** | **227650** | **AR** |
| **2** |  | **16:89839791** | **NM_000135** | **22** | **c.1902T>G**  **(p.Asp634Glu)** | **rs187300458** | **Het/WT/Het** | **0.0002** |  |  |  |
| 3 | PRSS1 | 7:142457375 | NM_002769 | 1 | c.40C>G  (p.Leu14Val) | rs747228052 | Het/Het/WT | 0.00001 | PCTT | 167800 | AD |
| 4 |  | 7:142457382 | NM_002769 | 1 | c.40+7A>T | rs796173487 | Het/Het/WT | / |  |  |  |
| 5 | POLR3A | 10:79741953 | NM_007055 | 28 | c.3718G>A  (p.Gly1240Ser) | rs1003620056 | Het/WT/Het | 0.00002 | HLD7; WDRTS | 607694; 264090 | AR; AR |
| 6 | KIF2A | 5:61657097 | NM_001098511 | 10 | c.901A>C  (p.Ile301Leu) | rs867833640 | Het/WT/Het | / | CDCBM3 | 615411 | AD |
| 7 | TTN | 2:179613239 | NM_133379 | 46 | c.13888C>G  (p.Leu4630Val) | rs139103966 | Het/Het/WT | 0.0024 | CMD1G; CMH9; LGMDR10; MFM9; SALMY; TMD | 604145 613765 608807 603689 611705 600334 | AD AD AR AD AR AD |
| 8 | ADCY5 | 3:123044288 | NM_183357 | 8 | c.1969G>A  (p.Ala657Thr) | rs775510502 | Het/Het/WT | 0.0002 | DSKOD; DSKOR; NEDHYD | 606703 619647 619651 | AD AR AR |
| 9 | DNAH9 | 17:11696841 | NM_001372 | 42 | c.8083G>C  (p.Glu2695Gln) | rs1336969191 | Het/WT/Het | / | CILD40 | 618300 | AR |
| 10 | KRT13 | 17:39661583 | NM_153490 | 1 | c.220G>A  (p.Gly74Arg) | Novel | Het/WT/Het | / | WSN2 | 615785 | AD |
| 11 | PLEC | 8:144997451 | NM_201384 | 31 | c.7057G>A  (p.Ala2353Thr) | rs1170367970 | Het/WT/Het | / | EBS5D; EBS5A; EBS5B; EBS5C; LGMDR17 | 616487 131950 226670 612138 613723 | AR AD AR AR AR |
| 12 | PRNP | 20:4680231 | NM_000311 | 2 | c.365T>G  (p.Val122Gly) | rs752571244 | Het/WT/Het | 0.0001 | GSD; CJD; GSD; HDL1; FFI | 137440 123400 137440 603218 600072 | AD AD AD AD AD |
| 13 | SHH | 7:155592897 | NM_001310462 | 4 | c.344C>T  (p.Ala115Val) | rs143502587 | Het/WT/Het | 0.0002 | HPE3; MCOPCB5; Schizencephaly; SMMCI | 142945 611638 269160 147250 | AD AD / AD |
| 14 | PROM1 | 4:16034977 | NM_006017 | 4 | c.459C>G  (p.Phe153Leu) | rs754270714 | Het/WT/Het | 0.0001 | CORD12; MCDR2; RP41; STGD4 | 612657 608051 612095 603786 | AD, AR AD AR AD |
| 15 | PER2 | 2:239169510- 239169513 | NM_022817 | 13 | c.1498_1500del  (p.Ser501del) | rs748056475 | Het/WT/Het | 0.00001 | FASPS1 | 604348 | AD |
| 16 | SLC7A9 | 19:33334760 | NM_014270 | 10 | c.1074+1G>T | Novel | Het/WT/Het | / | Cystinuria | 220100 | AD, AR |
| 17 | ADCY10 | 1:167805666 | NM_018417 | 23 | c.3190C>G  (p.Leu1064Val) | rs1329202333 | Het/WT/Het | / | HCA2 | 143870 | AD |
| 18 | KCNQ5 | 6:73843220 | NM_001160133 | 11 | c.1381G>A  (p.Val461Ile) | Novel | Het/WT/Het | / | MRD46 | 617601 | AD |
| 19 | PRX | 19:40902308 | NM_181882 | 7 | c.1951G>A  (p.Asp651Asn) | rs3814290 | Het/Het/WT | 0.0002 | CMT4F; CMT3 | 614895 145900 | AR AD, AR |
| 20 | NLRC4 | 2:32476322 | NM_021209 | 4 | c.611G>A  (p.Arg204His) | rs763335197 | Het/Het/WT | 0.00005 | FCAS4; AIFEC | 616115 616050 | AD  AD |
| 21 | PIEZO2 | 18:10718237 | NM_022068 | 34 | c.4876C>G  (p.Arg1626Gly) | rs533157733 | Het/Het/WT | 0.0002 | MWKS; DA3; DA5; DAIPT | 248700 114300 108145 617146 | AD AD AD AR |
| 22 | IRF2BPL | 14:77493375 | NM_024496 | 1 | c.761C>A  (p.Pro254His) | rs751106341 | Het/Het/WT | 0 | NEDAMSS | 618088 | AD |
| 23 | GREB1L | 18:18963579 | NM_001142966 | 3 | c.100C>T  (p.Arg34Trp) | rs559234950 | Het/Het/WT | 0.0002 | DFNA80; RHDA3 | 619274 617805 | AD  AD |
| 24 | PIKFYVE | 2:209190571 | NM_015040 | 20 | c.3036A>G  (p.Ile1012Met) | rs779842105 | Het/WT/Het | 0.00002 | CFD | 121850 | AD |
| 25 | SLC9A9 | 3:143100949 | NM_173653 | 13 | c.1477G>A  (p.Val493Met) | rs775249012 | Het/Het/WT | 0.0002 | AUTS16 | 613410 | / |
| 26 | F8 | X:154185372 | NM_000132 | 11 | c.1612G>A  (p.Asp538Asn) | rs1557281259 | Het/WT/Het | / | Hemophilia A | 306700 | XLR |
| 27 | PGK1 | X:77369291 | NM_000291 | 3 | c.167A>G  (p.Lys56Arg) | rs782543312 | Het/WT/Het | 0.0002 | PGK1 deficiency | 300653 | XLR |
| 28 | GPR101 | X:136113242 | NM_054021 | 1 | c.592G>C  (p.Val198Leu) | rs775145092 | Het/Het/WT | 0.0002 | PITA2 | 300943 | XL |
| 29 | BBS2 | 16:56536654 | NM_031885 | 8 | c.871G>A  (p.Gly291Ser) | rs528685215 | Het/WT/Het | 0.0009 | BBS2; RP74 | 615981 616562 | AR  AR |
| 30 | DPYD | 1:97915746 | NM_000110 | 14 | c.1774C>T  (p.Arg592Trp) | rs59086055 | Het/Het/WT | 0.0017 | DPD deficiency | 274270 | AR |
| 31 | ETFDH | 4:159603577 | NM_004453 | 3 | c.405+1G>A | Novel | Het/WT/Het | / | Glutaric acidemia II | 231680 | AR |
| 32 | MTHFD1 | 14:64877869 | NM_005956 | 3 | c.186+2_186+3insA | Novel | Het/Het/WT | 0.0042 | CIMAH | 617780 | AR |
| 33 | GNPTG | 16:1413033- 1413047 | NM_032520 | 11 | c.867_880del  (p.Lys290AlafsTer4) | rs1555452169 | Het/WT/Het | / | Mucolipidosis III gamma | 252605 | AR |
| 34 | ACP4 | 19:51294040 | NM_033068 | 3 | c.226C>T  (p.Arg76Cys) | rs1057519277 | Het/Het/WT | / | AI1J | 617297 | AR |
| 35 | CDAN1 | 15:43022830 | NM_138477 | 14 | c.2140C>T  (p.Arg714Trp) | rs80338696 | Het/Het/WT | 0.0004 | CDAN1A | 224120 | AR |
| 36 | DSP | 6:7566704 | NM_004415 | 8 | c.1034A>G  (p.Asp345Gly) | rs753855393 | Het/WT/Het | 0.0005 | ARVD8;  DCWHK;  DCWHKTA;  EBLA;  PPKS2;  SFWHS | 607450 605676 615821 609638 612908 607655 | AD AR AD AR AD AR |
| 37 | DYRK1A | 21:38884640 | NM_001396 | 12 | c.2098G>A  (p.Val700Ile) | rs540559440 | Het/WT/Het | 0.0006 | MRD7 | 614104 | AD |
| 38 | TSPAN12 | 7:120480171- 120480172 | NM_012338 | 3 | c.67-9delT | rs774111149 | Het/WT/Het | 0.0008 | EVR5 | 613310 | AD |
| 39 | HSPA9 | 5:137904633 | NM_004134 | 5 | c.516G>A  (p.Met172Ile) | rs544085290 | Het/WT/Het | 0.0039 | SIDBA4;  EVPLS | 182170 616854 | AD  AR |
| 40 | IARS | 9:95013035 | NM_002161 | 23 | c.2389G>A  (p.Asp797Asn) | rs199830006 | Het/Het/WT | 0.003 | GRIDHH | 617093 | AR |
| 41 | STT3A | 11:125489970 | NM_001278503 | 18 | c.2028T>A  (p.Asp676Glu) | rs771595862 | Het/WT/Het | 0.0002 | CDG1WAD;  CDG1WAR | 619714 615596 | AD AR |
| 42 | SARS | 1:109778650 | NM_006513 | 8 | c.1021T>C  (p.Phe341Leu) | rs143192294 | Het/WT/Het | 0.0088 | NEDMAS | 617709 | AR |
| 43 | SLC6A9 | 1:44467259 | NM_201649 | 9 | c.1222G>T  (p.Val408Phe) | rs149105213 | Het/WT/Het | 0.0002 | Glycine encephalopathy | 617301 | AR |
| 44 | MOGS | 2:74692301 | NM_006302 | 1 | c.74G>A  (p.Gly25Glu) | rs768041148 | Het/WT/Het | 0.0152 | CDG2B | 606056 | AR |
| 45 | RUSC2 | 9:35555393 | NM_014806 | 3 | c.2351G>A  (p.Arg784Gln) | rs137971910 | Het/WT/Het | 0.0086 | MRT61 | 617773 | AR |
| 46 | KATNB1 | 16:57785220 | NM_005886 | 6 | c.432+6G>A | rs116976731 | Het/Het/WT | 0.0199 | LIS6 | 616212 | AR |
| 47 | STAMBP | 2:74086480 | NM_006463 | 9 | c.1105C>T  (p.Pro369Ser) | rs761073632 | Het/Het/WT | 0.0003 | MICCAP | 614261 | AR |
| 48 | CIT | 12:120288058 | NM_007174 | 5 | c.436C>T  (p.Arg146Trp) | rs765923722 | Het/WT/Het | 0.0001 | MCPH17 | 617090 | AR |
| 49 | CEP152 | 15:49048668 | NM_014985 | 20 | c.2777A>T  (p.Glu926Val) | rs117557829 | Het/WT/Het | 0.0086 | MCPH9;  SCKL5 | 614852 613823 | AR AR |
| 50 | ATAD3A | 1:1461853 | NM_001170535 | 13 | c.1279G>A  (p.Glu427Lys) | rs759851546 | Het/WT/Het | 0.0014 | HAYOS;  PHRINL | 617183 618810 | AD,AR AR |
| 51 | FAT4 | 4:126337786 | NM_024582 | 6 | c.7018+9A>G | rs778593984 | Het/WT/Het | 0.00001 | HKLLS2;  VMLDS2 | 616006 615546 | AR AR |
| 52 | COL27A1 | 9:117028857 | NM_032888 | 33 | c.3406C>A  (p.Pro1136Thr) | rs199763065 | Het/WT/Het | 0.0012 | Steel syndrome | 615155 | AR |
| 53 | STRADA | 17:61784716 | NM_001003787 | 9 | c.644G>A  (p.Arg215His) | rs750512077 | Het/Het/WT | / | PMSE | 611087 | AR |
| 54 | NALCN | 13:101742191 | NM_052867 | 29 | c.3390+6C>T | rs200574014 | Het/WT/Het | 0.0031 | CLIFAHDD;  IHPRF1 | 616266 615419 | AD  AR |
| 55 | UNC80 | 2:210681642 | NM_032504 | 10 | c.1345C>T  (p.Arg449Cys) | rs1306849237 | Het/WT/Het | / | IHPRF2 | 616801 | AR |

Notes: HGVS, Human Genome Variation Society; P, patient; F, father; M, mother; Het, heterozygous; WT, wild type; MAF, minor allele frequency; MOI, mode of inheritance; AD, autosomal dominant; AR, autosomal recessive; dup, duplication; del, deletion; Ter, termination; fs, frameshift.

### Table S4. HPO searching

| **No.** | **Disease in HPO** | **Abbreviated name** | **OMIM** | **Inheritance** | **Gene** | **Loci** | **MONDO** |
| --- | --- | --- | --- | --- | --- | --- | --- |
| 1 | Centrifugal lipodystrophy | CLD | / | / | / | / | MONDO:0019552 |
| 2 | Ectodermal dysplasia with natal teeth, Turnpenny type | EDNT, Turnpenny type | 601345 | AD | / | / | MONDO:0011041 |
| 3 | Pachyonychia congenita 2 | PC2 | 167210 | AD | KRT17 | 17q21.2 | MONDO:0008174 |
| 4 | Gapo syndrome | GAPOS | 230740 | AR | ANTXR1 | 2p13.3 | MONDO:0009263 |
| 5 | Lenz-Majewski hyperostotic dwarfism | LMHD | 151050 | AR | PTDSS1 | 8q22.1 | MONDO:0007892 |
| 6 | Lipodystrophy, familial partial, type 2 | FPLD2 | 151660 | AD | LMNA | 1q22 | MONDO:0017230 |
| 7 | Cutaneous collagenous vasculopathy | CCV | / | / | / | / | MONDO:0017242 |
| 8 | Granddad syndrome | GS | 138920 | AD | / | / | MONDO:0007682 |
| **9** | **Wiedemann-Rautenstrauch syndrome** | **WDRTS** | **264090** | **AR** | **POLR3A** | **10q22.3** | **MONDO:0009910** |
| 10 | Amelo-onycho-hypohidrotic syndrome | AOHS | 104570 | AD | / | / | MONDO:0007095 |
| 11 | Lipodystrophy, familial partial, type 7 | FPLD7 | 606721 | AD | CAV1 | 7q31.2 | MONDO:0011714 |
| 12 | Ogden syndrome | OGDNS | 300855 | XLD, XLR | NAA10 | Xq28 | MONDO:0010457 |
| 13 | Microcephalic osteodysplastic primordial dwarfism, type 3 | MOPD3 | 210730 | AR | / | / | MONDO:0008873 |
| 14 | Steatocystoma multiplex-natal teeth syndrome | SMNTS | / | AD | / | / | MONDO:0008486 |
| 15 | Hypotrichosis-intellectual disability, Lopes type | HID Lopes type | / | AR | / | / | MONDO:0016414 |
| 16 | Spondylodysplastic Ehlers-Danlos syndrome | EDSSPD1 | 130070 | AR | B4GALT7 | 5q35.3 | MONDO:0034021 |
| 17 | Odontomicronychial dysplasia | OED | 601319 | AR | / | / | MONDO:0011034 |
| 18 | Pressure-induced localized lipoatrophy | PILL | / | / | / | / | MONDO:0019556 |
| 19 | Hypotrichosis simplex | HS | 605389 | AD | APCDD1 | 18p11.22 | MONDO:0018914 |
| 20 | Progeroid facial appearance with hand anomalies | PFAHA | 602249 | AD | / | / | MONDO:0011209 |

### Table S5. Evaluation by SpliceAI

| Chrom | Position | dbSNP ID | Ref | Alt | Filter | Format:Alleles\|Symbols\|DS_AG\|DS-AL\|DS_DG\|DS_DL\|DP_AG\|DP_AL\|DP_DG\|DP_DL |
| --- | --- | --- | --- | --- | --- | --- |
| 10 | 79743765 | rs1003620056 | G | A | 1000 | SpliceAI=A\|POLR3A\|0.00\|0.02\|0.00\|0.00\|-14\|5\|-2\|-15 |
| 10 | 79741953 | rs183347762 | C | T | 1000 | SpliceAI=T\|POLR3A\|0.00\|0.00\|0.14\|0.10\|-2\|0\|33\|0 |
| 16 | 89839791 | rs187300458 | T | G | 1000 | SpliceAI=C\|FANCA\|0.03\|0.00\|0.00\|0.00\|-1\|1\|-1\|-12 |
| 16 | 89828376 | / | C | CA | 1000 | SpliceAI=CA\|FANCA\|0.00\|0.00\|0.00\|0.00\|-10\|41\|-16\|-19 |

### Table S6. Nutrition factors for anemia

| **Items** | **Results** | **Unit** | **Reference** |
| --- | --- | --- | --- |
| Ferritin | 78.0 | ng/ml | 11-306.8 |
| Vitamin B12 | 213 | pmol/L | 133-675 |
| Vitamin D | 42.7 | ng/ml | >20 |
| Folate acid | 48.3 | nmol/L | 13.4-56.2 |

### Table S7. Mutations of thalassemia-related genes detected by SMRT sequencing

| **Names** | **Types (Numbers)** | **Abnormalities** |
| --- | --- | --- |
| ***Alpha Thalassemias*** | Deletions (30) | -α^3.7^, -α^4.2^, --^SEA^, --^THAI^, --^FIL^, --^MED-I^, --^MED-II^, --^11.1^, --^9.7^, -α^6.3^, -α^5.6^, -α^MAL3.5^, -α^2.7^, -α^2.4^, -α^2.8^, -α^1.2,^ -α^0.8^, -α^3.8^, -α^27.6^, Qinzhou type deletion |
|  | Recombinations (4) | ααα^anti3.7^, ααα^anti4.2^, Hkαα, antiHKαα |
|  | Mutations of HBA1 (80) | Hb Q-Thailand, Hb Hekinan II, Hb I, Hb Ube-2, Hb Beijing, Hb Shenyang, Hb Sichuan, CD68 (AAC>GAC), D11 (AAG>CAG), Hb Lille, Hb Asmsterdam-A1, Hb Owari, Initiation codon (-T), Initiation codon (A>G), Initiation codon (T>A) |
|  | Mutations of HBA2 (139) | Hb Quong Sze(QS), Hb Constant Spring(CS), Hb Westmead(WS), Hb G-Chinese, CD30(-GAG), CD8(-C), CD9(A>T), CD15(G>A), Fusion gene, CD31(G>A), -22C>T, CD40(-G), CD43-44(-C), CD49(-GC), CD61(A>T), Hb Dapu, Hb Debao, Hb Zurich-Albisrieden, Hb J-Broussais, Hb J-Wenchang-Wuming |
| ***Beta Thalassemias*** | Deletions (28) | (SEA)-HPFH, (Chinese)^G^γ^+^(^A^γδβ)0, Chinese I(εγδβ)0, Taiwanese, 3.5kb del, β7.3k del, δ87-β 16, β21.9kb del, Cantonese, Vietnamese HPFH, Yunnanese, HPFH-6, (^A^γδβ0)-thalassemia, Filipino del, β118kb del, β223 kb Chinese del |
|  | Mutations of HBB (340) | -28(A>G), -29(A>G), -30(T>C), -32(C>A), -90(C>T), -73(A>T), -50(G>A), CD31(-C), CD14/15(+G), CD17(A>T), CD27/28(+C), CD41/42(-TTCT), CD43(G>T), CD71/72(+A), IVS-I-1(G>A), IVS-I-1(G>T), HbE, IVS-I-5(G>C), IVS-II-654(C>T), Cap+43/+40(-AAAC), Initiation ATG>AGG, Cap+1(A>C), IVS-II-63(T>C), Cap+8(C>T), CD126(T>G), IVS-I-2(T>C) |

### Table S8. Results of SCGE assay

| **Group** | **HeadDNA**  **(%)** | **TailDNA**  **(%)** | **Tail length**  **(pix)** | **Tail moment**  **(TM)** | **Olive tail moment**  **(OTM)** |
| --- | --- | --- | --- | --- | --- |
| **Patient** | 92.59 | 7.41 | 16.63 | 3.17 | 2.71 |
| **Mother** | 98.02 | 1.98 | 8.92 | 0.50 | 0.60 |
| *p* value | 0.00 | 0.00 | 0.00 | 0.00 | 0.00 |
